# Informal mhealth at scale in Africa: Opportunities and challenges

**DOI:** 10.1016/j.worlddev.2020.105257

**Published:** 2021-04

**Authors:** Kate Hampshire, Tawonga Mwase-Vuma, Kassahun Alemu, Albert Abane, Alister Munthali, Tadesse Awoke, Simon Mariwah, Elita Chamdimba, Samuel Asiedu Owusu, Elsbeth Robson, Michele Castelli, Ziv Shkedy, Nicholas Shawa, Jane Abel, Adetayo Kasim

**Affiliations:** a Department of Anthropology, Durham University, Durham DH1 3LE, UK; b University of Malawi, Malawi; c University of Gondar, Ethiopia; d University of Cape Coast, Ghana; e University of Hull, UK; f University of Newcastle, UK; g University of Hasselt, Belgium; h Research Triangle Institute, United States; i Durham University, UK

**Keywords:** Mobile health, Informal mhealth, Community health workers, Ghana, Malawi, Ethiopia

## Abstract

The extraordinary global growth of digital connectivity has generated optimism that mobile technologies can help overcome infrastructural barriers to development, with ‘mobile health’ (mhealth) being a key component of this. However, while ‘formal’ (top-down) mhealth programmes continue to face challenges of scalability and sustainability, we know relatively little about how health-workers are using their own mobile phones *informally* in their work. Using data from Ghana, Ethiopia and Malawi, we document the reach, nature and perceived impacts of community health-workers’ (CHWs’) ‘informal mhealth’ practices, and ask how equitably these are distributed. We implemented a mixed-methods study, combining surveys of CHWs across the three countries, using multi-stage proportional-to-size sampling (N = 2197 total), with qualitative research (interviews and focus groups with CHWs, clients and higher-level stake-holders). Survey data were weighted to produce nationally- or regionally-representative samples for multivariate analysis; comparative thematic analysis was used for qualitative data. Our findings confirm the limited reach of ‘formal’ compared with ‘informal’ mhealth: while only 15% of CHWs surveyed were using formal mhealth applications, over 97% reported regularly using a personal mobile phone for work-related purposes in a range of innovative ways. CHWs and clients expressed unequivocally enthusiastic views about the perceived impacts of this ‘informal health’ usage. However, they also identified very real practical challenges, financial burdens and other threats to personal wellbeing; these appear to be borne disproportionately by the lowest-paid cadre of health-workers, especially those serving rural areas. Unlike previous small-scale, qualitative studies, our work has shown that informal mhealth is already happening *at scale*, far outstripping its formal equivalent. Policy-makers need to engage seriously with this *emergent health system*, and to work closely with those on the ground to address sources of inequity, without undermining existing good practice.

## Highlights

• Community health-workers across Africa use mobile phones ‘informally’ in their work.
• Informal mhealth is an emergent phenomenon, used to bridge gaps in formal provision.
• Informal mhealth is happening at scale, far outstripping its formal equivalent.
• Informal mhealth is inherently responsive to local needs and contingencies.
• But it carries hidden costs (financial and other) which are inequitably distributed.

## 1 Introduction

The growth of global digital connectivity over the last two decades has been extraordinary. In 2019, there were 8.3 billion active mobile phone subscriptions (approximately 1.08 *per capita*), of which 6.7 billion were held in low- and middle-income countries (LMICs) (ITU, n.d.). An estimated 96.6% of the world’s population, and 96.2% of people living in LMICs, are covered by a mobile-cellular network, with 3G connectivity now reaching the vast majority (93.0% globally, 92.0% in LMICs) (ITU, n.d.) It is no exaggeration to say that mobile phones have impacted the lives of almost everyone on the planet, but the transformation has been most remarkable in LMICs, where access to fixed-line telecommunications had been limited.

From its earliest days, the ‘digital revolution’ has been seen to offer a route to overcoming (or ‘leapfrogging’) infrastructural barriers to development, especially for poor, rural and otherwise marginalised communities (Steinmueller, 2001). Twenty years on, accelerating adoption of mobile technology in Africa appears to have driven economic growth (Donou-Adonsou et al., 2016, Haftu, 2019). However, the initial optimism has been somewhat tempered by the emergence of new digital divides and the recognition that the benefits of these technologies can be patchy, unsustained and inequitable. A prominent World Bank report (2016:1) noted that, although “in many instances, digital technologies have boosted growth, expanded opportunities, and improved service delivery”, “their aggregate impact has fallen short and is unevenly distributed”. The report noted that digital dividends were unevenly distributed both *between* and *within countries* with lower-income groups, women, older people and rural populations disproportionately excluded (Ibid 2016: 7). A recent WHO Guideline (2019:v) also highlighted the need to “make sure that innovation and technology helps to reduce the inequities in our world, instead of becoming another reason people are left behind.”

This paper focuses on one particular form of ‘digital development’: ‘mobile health’ (mhealth), enthusiastically embraced by governments and international donors as a means to improve health service delivery and achieve Universal Health Coverage (UHC) goals (Mehl and Labrique, 2014, World Health Organization, (2011)). There is general agreement that mhealth (and its cousins, telemedicine and e-health^1^) can be particularly beneficial for delivering care in remote, rural areas with sparse physical infrastructure (World Health Organization, 2019). As such, applications designed to support rural community health-workers (CHWs) have been a particular focus. These include (*inter alia*): case-management and referral tools for CHWs; messaging services to clients (reminders, advice, etc.); data capture and management systems; stock management and other logistical applications; and resources for staff training, monitoring and support (Aranda-Jan et al., 2014, Marcolino et al., 2018).

Several recent systematic reviews have sought to assess the impacts of ‘official’ mhealth programmes in LMIC settings (Aranda-Jan et al., 2014, Feroz et al., 2017, Mekonnen et al., 2019, Marcolino et al., 2018, Watkins et al., 2018), reaching similar conclusions: that, while the evidence base remains limited, some initiatives appear to have produced positive changes in health behaviours (e.g. clinic attendance; adherence to long-term medication) and outcomes (e.g. decreased viral load), especially in low-income, rural and otherwise “hard-to-reach” communities. In another recent review, Odendaal et al. (2020) concluded that mhealth had led to (mostly) better communication practices between health-workers and with patients.

However, as with other forms of ‘digital development’, the costs and benefits of mhealth are not necessarily evenly distributed. While the transformative potential may be greatest in rural areas, problems of poor network connectivity, electricity supply and other logistical challenges disproportionately affect those same areas (World Health Organization, 2019: 35). Moreover, Jennings and Gagliardi (2013) have warned that some programme designs (even those targeting women specifically, such as maternal/newborn health initiatives) may inadvertently reinforce the gender digital divide. Scalability and sustainability also remain major challenges. An over-reliance on donor funding without effective integration into national health systems has resulted in the phenomenon of ‘pilotitis’ (Huang, Blaschke, & Lucas, 2017): the repeated launching of small-scale projects without a clear route towards scale or sustainability. The consequence is what Whyte, Meinert, and Twebaze (2013) have called ‘projectified landscapes of care’, with access to healthcare becoming highly fragmented and contingent on being in the right place at the right time, or knowing the right people – the antithesis of Universal Health Coverage.

While the potential of what we might call ‘*formal mhealth*’ (top-down programmes initiated by governments or donors) has therefore yet to be fully realised, we know relatively little about what health-workers are doing *with their own phones*, and with what consequences. The vast majority of research and policy interest has focused on formal initiatives but, based on subscription data, we can reasonably assume that most health-workers worldwide own a mobile phone. A few, mostly small-scale, qualitative, studies have investigated what we have called ‘*informal mhealth*’ (Hampshire et al., 2015; Hampshire et al., 2017) – the ‘spontaneous’ or ‘bottom-up’ use of phones by practitioners and/or patients^2^ for healthcare purposes; we review this literature below. This current paper is, we believe, one of the first to research the phenomenon on a larger scale. Using survey data from Ghana, Ethiopia and Malawi, complemented by multi-sited qualitative research, we address four key questions:

(1) What is the relative reach of ‘formal’ and ‘informal’ mhealth among community health-workers (CHWs) across the three countries?
(2) How do CHWs use mobile phones informally in their work?
(3) What are the perceived impacts of ‘informal mhealth’?
(4) How are these practices and perceived impacts distributed, between and within countries?

## 2 Background: technological appropriation and the phenomenon of ‘informal mhealth’

In a landmark study, tracing the spread of mobile phones in Jamaica, Horst and Miller (2006) made the important observation that digital technologies are not simply adopted passively as they move around the world. Instead, they are appropriated, co-opted and used in ways that were not necessarily intended (or desired) by manufacturers. A wealth of recent scholarship has documented the appropriation of mobile phones in many different contexts (see overviews by Ling and Horst, 2011, Oreglia and Ling, 2018). For example, the phenomenon of phone-based Keitai novels in Japan (Nishimura, 2011), or the proliferation of new language forms that SMS texting has generated in Senegal (Lexander, 2011), exemplify the inter-weaving of the local and the global in new cultural forms. The work of Sey (2011) in Ghana and de Souza e Silva, Sutko, Salis, and de Souza e Silva (2011) in Brazil has shown how mobile phones have become incorporated into and shaped almost every aspect of social life, simultaneously challenging and reinforcing existing structures and hierarchies. For other more recent examples, see Aricat and Ling (2018) re Burmese fishers; Larsson and Svensson (2018) re female market traders in Uganda; and Djohy, Edja, and Schareika (2017) re pastoralists in northern Benin.

A small strand of this literature has examined the ‘appropriation’ of mobile phones for health-related purposes. One of the earliest contributions came from Patricia Mechael’s (2009) account of ‘organic mhealth’ practices in Egypt in the early 2000s, with both health professionals and ‘lay people’ using phones to bridge gaps in healthcare provision. Since then, a few other researchers have documented similar practices across a range of settings. Examples include: Oliver, Geniets, Winters, Rega, & Mbae's (2015) study of CHWs in Kenya, who used personal mobile phones to help manage heavy workloads and resource constraints; our own previous work (Hampshire et al., 2017) on informal mhealth practices of CHWs in Ghana and Malawi; and Watkins, Goudge, Gómez-Olivé, and Griffiths' (2018) account of ‘bottom-up digital initiatives’ by health professionals and patients in South Africa. Also in South Africa, Mars and Scott (2017) described the rise of ‘spontaneous telehealth’, whereby junior doctors would share images of dermatological conditions with specialist consultants for advice. Williams and Kovarik (2018) reported a similar phenomenon in Botswana, while Ling, Poorisat, and Chib (2020) recently wrote about the informal use of phones by health-workers in Thailand for patient referral.

Taken as a whole, this body of work has been important in highlighting the creativity and commitment of health-workers in many different settings who use personal phones ‘informally’, ‘spontaneously’ or ‘organically’ to plug gaps in formal health systems. As Ling et al. (2020) show, these bottom-up, ‘extra-systemic’ practices may become interwoven into existing (formal) healthcare structures, to produce more flexible, responsive and workable systems. However, this work has also revealed a range of potential costs and challenges, from failures in telecommunications infrastructure, to financial burdens, to increased workload demands and associated threats to wellbeing (Mechael, 2009, Oliver, Geniets, Winters, Rega, & Mbae, 2015, Hampshire et al., 2017).

These small-scale, qualitative studies have provided important insights into practices and experiences on the ground; however, their design means they can tell us little about the *scale* of informal mhealth and its impacts beyond the few communities investigated. To our knowledge, only one group of researchers has investigated informal mhealth systematically, on a larger scale. Using household panel data from India and China, Haenssgen and colleagues (Haenssgen and Ariana, 2017, Haenssgen, 2018, Haenssgen, 2019) reported that, in the absence of specific mhealth interventions, mobile phones were changing the ways that people sought healthcare. However, while ‘informal’ phone use appeared to enhance healthcare access *overall* (Haenssgen & Ariana, 2017), it risked *exacerbating*, rather than ameliorating, health inequalities (Haenssgen, 2019). In some cases, poorer households with limited digital access experienced an *absolute worsening* of provision, as healthcare became increasingly digitally mediated (Haenssgen, 2018).

The work of Haenssgen and colleagues is thus crucial in moving us beyond localised case studies to considering the wider patterning of practices and experiences, including the possibility that informal mhealth might systematically benefit or exclude particular groups. However, Haenssgen et al. were only able to look at the demand side – those seeking care. The study we report on here is the first, we believe, to apply a similarly rigorous, quantitative approach to service providers – in this case community health-workers. Building on the our own and others’ previous qualitative work, we take the next important step by reporting on mhealth practices across large, representative samples of CHWs in three African countries, and analysing variation between and within countries, by gender, age and rural/urban location, the most widely-reported correlates of digital exclusion (World Bank, 2016, Porter et al., 2016, Porter et al., 2020)^3^. Our data suggest that informal mhealth constitutes a *large-scale emergent health system*, far outstripping the reach of its ‘formal’ equivalent, with urgent policy implications.

## 3 Context: Community health-workers and mhealth in Ghana, Malawi and Ethiopia

Ghana, Malawi and Ethiopia were purposively selected for this study because all three countries have national CHW and ehealth/mhealth strategies in place, but with substantial variation in programming and coverage: Table 1.

**Table 1:** Key characteristics of each country.

	Ghana	Ethiopia	Malawi
Total population (millions)	29.8	109.2	18.1
% rural population	44%	79%	83%
Per capita GNI (USD)	4,650	2,010	1,310
Life expectancy at birth (years)	63	66	63
Under-five mortality rate (per 1000 live births)	48	55	50
Mobile phone subscriptions (per 100 people)	137.5	36.2	39.0
Internet users (per 100 people)	39.0	18.6	13.8
Approx. number of CHWs^a^	17,400	38,000	10,000
Approx. population per CHW	1,700	2,900	1,800
Number of current (live) mhealth initiatives^b^	30	9	32

*Notes:*

1. Sources: All data (except ^b^) from World Bank Open data 2018: https://data.worldbank.org/.

2. ^a^CHNs in Ghana, HEWs in Ethiopia, HSAs in Malawi.

3. ^b^Data from GSMA Global mHealth Deployment Tracker: https://www.gsma.com/mobilefordevelopment/m4d-tracker/mhealth-deployment-tracker/.

### 3.1 Community Health-Worker programming

Community Health-Workers have long been promoted by the World Health Organisation as a pragmatic solution to address gaps in human resources for health. The World Health Organization, (2018:22) definition of CHWs, as “health workers based in communities […] who are either paid or volunteer, who are not professionals, and who have fewer than two years training but at least some training, if only for a few hours” covers a wide variety of personnel with very different levels of training, remuneration and roles (Olaniran, Smith, Unkels, Bar-Zeev, & van den Broek, 2017). Here, we focus on the largest cadre in each country charged with delivering basic promotive, preventive and curative services at community level: Community Health Nurses (CHNs) in Ghana; Health Surveillance Assistants (HSAs) in Malawi; and (all female) Health Extension Workers (HEWs) in Ethiopia. All of these are government employees; at the time of fieldwork, they received monthly salaries in the region of 150 GBP (1100 GHS) in Ghana, 110 GBP (500 ETB) in Ethiopia and 100 GBP (100,000 MKW)^4^ in Malawi, plus allowances for training, etc. Salary differences partly reflect variation in training and professional status: Ghanaian CHNs are qualified nurses (thus actually contravening the WHO definition) with two years’ post-secondary training; Ethiopian HEWs receive a year’s post-secondary training; while initial HSA training in Malawi lasts just 12 weeks. Coverage also varies substantially between the three countries: Table 1.

In each country, salaried CHWs are supported by unpaid volunteers: Community Health Volunteers (CHVs) in Ghana; Village Health Committees (VHCs) and others in Malawi; and the all-female Health Development Army (HDA) in Ethiopia. Below, we use the term ‘Community Health-Workers’ (CHWs) to refer specifically to the salaried employees who are the focus of this study, while ‘volunteers’ refers to those who support them without pay.^5^

### 3.2 mhealth strategies and initiatives

National eHealth/mhealth strategies were launched in 2010 in Ghana (Ghana Ministry of Health, 2010) and 2014 in Malawi and Ethiopia (Government of Malawi, 2014, Government of Ethiopia, 2014). However, mobile phone coverage and the reach of ‘formal’ mhealth programmes varies substantially across the three countries. Ghana has by far the highest mobile phone penetration, with an estimated 137 subscriptions per 100 people, compared with just 35–40 per hundred people in Ethiopia and Malawi; levels of internet usage are also greatest in Ghana.

Of the three countries, formal mhealth is most strongly established in Malawi, with 30 currently-active programmes functioning at different scales, including the highly-acclaimed SMS-based C-Stock platform for managing medicine supplies, operating at its height in 24 of Malawi’s 28 Districts (Shieshia et al., 2014). Ghana also has a relatively large number of current projects (32), but coverage has tended to be more limited, even for some of the higher-profile initiatives like MoTech (Mobile Technology for Community Health), launched in 2010 to interface between pregnant mothers and ante-natal services but never achieving the intended level of scale-up (LeFevre et al., 2017, Willcox et al., 2019). Ethiopia has by far the fewest currently-active programmes (8); mostly small-scale initiatives targeting HEWs (e.g. Steege et al., 2018, Thomsen et al., 2019). Overall, with one or two notable exceptions, mhealth in Ghana, Malawi and Ethiopia has faced the same challenges of sustainability and scalability those encountered in other locations, described above.

## 4 Methods

### 4.1 Study design and overview

Data reported here were collected in Ghana, Malawi and Ethiopia between May 2018 and September 2019 as part of the IMAGINE project (Informal Mhealth in Africa: Grassroots Innovation and Networks)^6^. Fieldwork proceeded in three main phases in each country, with large-scale questionnaire surveys of CHWs (N = 2197) sandwiched between two phases of multi-sited qualitative work.

### 4.2 Study locations

The three countries were selected on the basis of variation in formal mhealth coverage and CHW programming (see above). In each country, the aim was to maximise geographical coverage within the project’s time and budgetary constraints. In Malawi (the smallest country by population and area), it was possible to work in 27 of the 28 Districts^7^, covering all three Regions (Northern, Central and Southern). This level of national coverage was not feasible in the other two countries. In Ghana, three of the (then) ten Regions^8^ (Central, Brong Ahafo, and Northern Region) were purposively selected to represent the country’s three principal agro-ecological zones (mapping onto broad ethnic categories). In Ethiopia, we purposively selected the two largest Regions (out of nine): Amhara and Oromia which, between them, comprise c.52% of Ethiopia’s total population and 65% of HEWs. Sampling within regions was specific to the fieldwork phase: see below.

### 4.3 Phase 1: exploratory qualitative research

The aim of Phase One (May–July 2018) was to establish the key parameters of mhealth practices and experiences in each country, and to inform questionnaire design for Phase Two.

In Ghana and Malawi, focus group discussions (FDGs) were conducted with CHWs at two urban and four rural health facilities in each of three Districts (one per study Region, selected randomly), i.e. eighteen FDGs per country in total, with an average of 7–8 participants per group. In Ethiopia, the greater geographical dispersion of HEWs made FGDs impractical at this stage; instead, 36 individual semi-structured interviews (SSIs) were conducted with HEWs across rural and urban locations in Amhara (N = 19) and Oromia (N = 17) Regions.

Topic guides for FGDs and SSIs were similar, covering six thematic areas: CHW roles, formal mhealth, informal mhealth, phonelessness, evaluations of experiences, and ‘ways forward’; each with indicative questions and prompts. FGDs were facilitated in local languages by two in-country researchers (one facilitator and one note-taker), while SSIs were conducted one-to-one. On average, FDGs lasted around 1–1.5 h, while SSIs took approximately 30–40 min. Audio recordings were made with participants’ explicit permission; these, along with hand-written notes, were subsequently transcribed and translated into English, keeping key terms in the original language. Analysis followed the principles of Comparative Thematic Analysis (Braun & Clarke, 2006): two of the authors familiarised themselves thoroughly with the material, reading and re-reading transcripts; an initial set of inductive codes was agreed and applied to the data. Codes were then grouped into themes and reviewed, generating a thematic ‘map’ or framework for analysis.

Interviews were also conducted at this stage with higher-level stake-holders, representing relevant public, private and voluntary-sector organisations in each country (N = 7 in Ghana, N = 5 in Ethiopia, N = 15 in Malawi). The aim of these interviews was to provide additional contextual information, helping us to situate and cross-check information provided by the CHWs. Participants included national/local representatives from Ministries of Health and other major healthcare providers (including CHAG in Ghana and CHAM in Malawi^9^), NGOS and donors implementing mhealth programmes, and health-workers’ associations.

### 4.4 Phase Two: CHW survey

The aim of Phase Two was to ascertain the scale, nature and variation in mhealth practices and experiences in each country, through questionnaire surveys of CHWs.

#### 4.4.1 Questionnaire design and piloting

Questionnaire design was closely informed by Phase One findings, to ensure comprehensive coverage of all relevant practices and experiences. An initial questionnaire was piloted and re-piloted sequentially in several sites per country^10^, with amendments made after each stage. The final version contained 95 items, grouped under 6 themes: background information (on the individual and the facility); phone ownership, access and usage; formal and informal mhealth usage; perceived impacts); and possible ‘ways forward’. Questions were mostly closed-response, with respondents selecting from a list of pre-specified options, derived from Phase 1 fieldwork and refined following piloting, and (where applicable) an ‘other’ category^11^. The final version of the questionnaire was translated into local languages (with back translation to check quality); it took around 20–30 min to administer.

#### 4.4.2 Sampling

Target survey sample sizes were: 480 in Ghana and Malawi, and 960 in Ethiopia, based on a power calculation assuming 5% margin of error in estimating work-related phone usage and a survey design effect of 25%, including missing data^12^. Multistage sampling of CHWs was employed in all three countries, using probability-proportional-to-size (PPS) allocation. The sampling scheme was adapted for each country to account for the differences in total size/population, administrative structures and geographical distribution of CHWs. As noted above, in Malawi, we were able to sample across the whole country (except for Likoma Island), while we purposively selected three out of ten Regions in Ghana and two out of nine in Ethiopia. In Malawi, health facilities were stratified as Government- or CHAM-operated, with over-sampling of CHAM health facilities to ensure adequate representation; health facilities were then selected in each District proportional to the total number in that District. In Ghana, a random sample of districts was drawn within each selected region; health facilities were then sampled on a proportional-to-size basis. A similar approach was taken in Ethiopia Regions, but with rural–urban stratification of districts within each region. Further details of the sampling procedures are shown in Table 2. The final survey sample size was 2197 CHWs (598 in Ghana, 1019 in Ethiopia, and 580 in Malawi. Response rates were: 99.6% (Ghana), 100% (Ethiopia), and 96.7% (Malawi)^13^.

**Table 2:** Survey design and sampling.

	GHANA	ETHIOPIA	MALAWI
Target sample size	480	960	480
Selection of Regions	3 regions out of 10 (Central, North, Brong Ahafo) purposively selected	2 regions out of 9 (Amhara & Oromia) purposively selected	All 3 regions included.
Stratification	N/A	Stratification of Districts within each Region into urban and rural: Amhara: 38 urban Districts; 138 rural Districts. Oromia: 48 urban Districts; 187 rural Districts.	Stratification of health facilities into those run by government (75%) and those run by CHAM (Christian Health Association of Malawi, 25%)
Sampling stage 1: Districts	Random selection of 5 Districts within each Region: Central: 5/20 Districts (25%) BA: 5/27 Districts (18.5%) Northern: 5/26 Districts (19.2%)	Random selection of 20% of Districts (woreda) within each stratum: Amhara urban: 8/38 Districts; Amhara rural: 28/132 Districts; Oromia urban: 10/48 Districts; Oromia rural: 37/187 Districts.	27/28 Districts selected (excluding Likoma Island).
Sampling stage 2: Health Facilities (proportional-to-size allocation)	Random sampling of health facilities in each selected District, to achieve proportional-to-size allocation of CHNs. Sampling frame: lists from Regional and District Directorates. Total of 3530 CHNs in the 3 Regions (1332 in Central Region, 1223 in Brong Ahafo, 975 in Northern Region). Target allocation of 600 CHNs: Central Region: 226 Brong Ahafo: 208 Northern Region: 166 Total sample: 308 health facilities Central: 97 (23 urban; 74 rural); BA: 113 (32 urban; 81 rural); Northern: 98 (29 urban; 69 rural).	Random sampling of health centres within each selected District, to achieve proportional-to-size allocation of HEWs. Sampling frame: Regional Health Bureaus records of health facilities. Total of 23,590 HEWs in the 2 Regions (7893 in Amhara, 15,697 in Oromia). Target allocation of 1000 HEWs: Amhara urban: 34 Amhara rural: 301 Oromia urban: 60 Oromia rural: 605. Total: 233 health centres: Amhara urban: 5 HCs (20%), Amhara rural: 106 HCs (29%), Oromia urban: 6 HCs (30%), Oromia rural: 116 HCs (32%).	Random sampling of health facilities in each District on a proportional-to-size basis. Sampling frame: Ministry of Health lists of (total of 977 of Government and CHAM health facilities across the three Regions: Northern 167; Central 364; Southern 446). Target allocation of N = 150 health facilities: Northern: 31 Central: 54 Southern: 65 This was further broken down by district, with proportional to size allocation of health facilities in each District. Total sample: 150 facilities. Northern: 31 (7 urban & 24 rural). Central: 54 (11 urban & 43 rural). Southern: 65 (18 urban & 47 rural).
Sampling stage 3 (CHWs)	Every CHN available for interview at time of visit: **Total: 598 CHNs** Central: 189 (31 urban; 158 rural) BA: 207 (70 urban; 137 rural) Northern: 202 (71 urban; 131 rural).	All HEWs working at health posts clustered under each HC. **Total: 1019 HEWs** Amhara urban: 29 HEWs Amhara rural: 331 HEWs Oromia urban: 114 HEWs Oromia rural: 545 HEWs	Random selection of up to 2 male and 2 female HSAs per facility, present at visit. **Total: 580 HSAs** Northern: 117 (27 urban; 90 rural). Central: 212 (44 urban; 168 Rural). Southern: 251 (74 urban; 177 Rural).
Overall response rate	99.6%	100%	96.7%
Use of replacements	No	No	No
Use of re-visits	No	None required	Two re-visits made.
Construction of survey weights	Survey weights were approximated as the inverse probability of sampling a health facility within each region. The limitation of this approach is that we imposed equal weights in participants from the same region, since there was no denominator data to calculate weights for the individual CHWs. However, it is a better compromise than using unweighted data for analysis.	Survey weight was calculated by taking the inverse of the product of the probabilities of selection at each stage of the sampling scheme: 1. Probability of selecting each zone (selected Zone/Total Zones). 2. Probability of selecting districts within the Zone (selected districts/total districts). 3. Probability of selecting HC within the selected districts	Survey weight was calculated by taking the inverse of the product of the probabilities of selection at each stage of the sampling scheme: 1. Probability of selecting a particular Government or CHAM health facility within a district; 2. Probability of selecting an HSA within a sampled health facility..

#### 4.4.3 Data collection, management and analysis

The survey was administered face-to-face (October 2018 to March 2019) by trained researchers, using computer-assisted personal interview software (CSPro 7.2) on Android-based tablets. The lead researcher in each country supervised data collection closely, checking the data on a daily basis for possible errors, inconsistencies and missing data. Where possible, these were corrected by re-visiting the participant the same day. Data were uploaded every week (more often where internet connectivity permitted) to a secure central server at Durham University (UK), where additional random spot checks were conducted for quality. Survey data were exported from CSPro to Stata/IC (version 15.1 for Mac) and SPSS (version 26) software for cleaning, management and analysis.

The survey data were weighted to produce a nationally-representative dataset for Malawi, and regionally-representative datasets for Ghana and Ethiopia. The survey design was incorporated in all analyses with appropriate weighting, defined as the inverse of sampling probabilities for each CHW. However, survey weights for CHWs in Ghana were assumed to be the same for CHWs from the same region due to lack of data on total number of CHWs in each surveyed health facility.

Descriptive statistics were produced initially, using cross-tabulations by country, and a meta-regression method to compare between countries. Survey linear regression analysis was then performed on the data for each country separately, with gender, age and rural/urban location as covariates. Survey logistic regression models were used for binary outcomes and survey linear regression for continuous outcomes, assuming Gaussian distributions. Cross-tabulation showed interactions between gender and location in Malawi and Ghana (see Table 5) but the study was not powered sufficiently to include interaction effects in the regression models. We made the decision to analyse the data for each country separately, rather than in aggregate, because of the different sampling techniques and the different geographical/demographic distributions of CHWs^14^ (see Table 3). We then used a meta-analytic method to obtain aggregated data across all countries, after data from each country had been weighted according to survey design and sampling techniques; however, the heterogeneity between countries limits the utility of the meta-analysis^15^.

**Table 3:** Survey Sample characteristics (valid unweighted and weighted percentages).

	Ghana (N = 598)		Ethiopia (N = 1019)		Malawi (N = 580)	
	Unweighted	Weighted	Unweighted	Weighted	Unweighted	Weighted
Gender^1^						
Female	74.7%	73.7%	100%	100%	44.3%	50.7%
Male	25.3%)	26.3%	0		55.7%	49.3%
Age^2^ (median, range)	30y (22-59y)		28y (19-45y)		40y (28-59y)	

Region^3^						
Region 1	31.6%	27.3%	35.3%	32.8%	20.0%	12.3%
Region 2	34.6%	32.7%	64.7%	62.7%	36.6%	40.2%
Region 3	33.8%	40.0%			43.4%	47.6%

Location						
Urban	28.8%	29.6%	14.0%	6.8%	25.5%	41.2%
Rural	71.2%	70.4%	86.0%	93.2%	74.5%	72.9%

Facility base type^4^						
Hospital/polyclinic	11.1%	10.9%			18.8%	27.1%
Rural hosp/health centre	34.9%	3.5%			81.2%	72.9%
Health post	54.0%	7.5%	100%	100%	(47.9%)^4^	

Job title						
Community Health Nurse	89.1%	89.1%				
Comm Mental Health Nurse	3.3%	3.5%				
Other nurse/midwife	6.8%	7.5%				
Health Extension worker			100%	100%		
Health Surveillance Assist					75.7%	75.2%
Senior HSA					34.2%	24.8%

Education level (highest)						
<Secondary completion	0	0	0.1%	0.1%	25.0%	25.7%
Secondary completed	0	0	85.3%	91.6%	72.8%	71.8%
Certificate/Diploma/Degree	100%	100%	14.2%	8.5%	2.2%	2.5%

*Notes:*

1. In Ethiopia, all Health Extension Workers are female.

2. In Malawi, recruitment of HSAs has been limited since 2007, hence the older age distribution.

3. Coding of Regions: Ghana: 1 = Central; 2 = Brong Ahafo, 3 = Northern. Ethiopia: 1 = Amhara, 2 = Oromia. Malawi: 1 = Northern, 2 = Central, 3 = Southern

4. In Malawi, HSAs whose communities are ‘hard to reach’ also operate a Village Clinic, regardless of the level of health facility to which they are formally attached.

### 4.5 Phase Three: follow-up qualitative research

In the final phase of fieldwork (June–August 2019), another round of 42 FGDs were convened with CHWs (N = 6–9 per country) and community members (N = 6–9 per country) to share and discuss the survey findings, and to deepen our contextual understanding. Again, these were conducted in rural and urban settings across all study Regions in each country, but in different locations to those used in Phase One. This time, there was some variation in topic guides between the three countries, to account for differences in survey findings. The procedures for convening the FGDs; recording, transcribing and translating the material; and the analytical approach, were identical to Phase One.

### 4.6 Ethics, positionality and implications for data quality

Ethical approvals for the project were given by national and university review boards in each country; permissions were also sought from Regional and District level authorities. Researchers provided and read aloud information sheets to prospective participants, who gave fully informed consent (in writing or verbally, according to preference).^16^ Participants were assured that participation in the research was entirely voluntary and that they could withdraw at any point. No financial incentives were offered, although focus group discussants were provided with light refreshments. All transcripts were anonymised and uploaded to a secure GDPR-compliant central database; original recording/notes were then destroyed.

This was a highly collaborative project with a team of 11 investigators from five countries (all authors on this paper), who worked closely together throughout, from initial study design through to analysis and drafting research papers. Fieldwork in each country was carried out by experienced teams of local researchers overseen by country leads. Each country team took primary responsibility for analysing their country’s data and contributing to overall comparative analysis.

As far as possible, we tried to ensure that fieldworkers shared an ethno-linguistic background with research participants to facilitate communication. Nonetheless, there were clear status/income differences between PhD-holding academics and study participants with limited educational or economic opportunities. Researchers sought to minimise these differences through the way they dressed and comported themselves. Study participants appeared to feel at ease, responding thoughtfully and frankly to our questions. We are aware that healthcare delivery is not politically neutral; prevailing political discourses may shape what health-workers are prepared to *say* (despite assurances of confidentiality), or even *think* (for a very relevant example, see, Østebø, Cogburn, & Mandani, 2018, on the silencing of criticism of Ethiopia’s Health Extension Programme). While our study participants appeared to speak freely and were often quite critical of government programmes, we cannot rule out the possibility of ‘social desirability bias’ (Bergen & Labonté, 2020); for example, CHWs being more willing to discuss positive examples of phone use rather than detrimental ones. Moreover, although we explained very clearly that we were academic researchers rather than policy-makers, it is conceivable that some respondents might have exaggerated estimates of expenditure, etc., in order to try to secure assistance.

## 5 Results

### 5.1 Participant characteristics

Demographic characteristics of the study participants are shown in Table 3, Table 4. There is notable between-country variation in the survey sample, reflecting the health systems differences outlined above. In Ethiopia, all HEWs are female, whereas both genders were represented (in different proportions) in Ghana and Malawi. Average age was highest in Malawi, where the last major HSA recruitment drive happened in 2007. Educational level was highest in Ghana and lowest in Malawi, reflecting the different CHW training requirements in each country.

**Table 4:** Qualitative samples: raw numbers of interviewees and focus group participants (CHWs, clients and volunteers).

	Ghana		Ethiopia		Malawi	
	CHWs	Clients & volunteers	CHWs	Clients & volunteers	CHWs	Clients & volunteers
Gender^1^						
Female	60	67	63	10	35	75
Male	20	4	–	22	57	36

Region^2^						
Region 1	25	19	23	13	18	27
Region 2	30	20	40	19	25	26
Region 3	25	32	–	–	49	58

Location						
Urban	27	22	16	14	33	40
Rural	53	49	47	18	59	71
TOTALS	80	71	63	32	92	111

*Notes:*

1. In Ethiopia, all Health Extension Workers are female.

2. Coding of Regions: Ghana: 1 = Central; 2 = Brong Ahafo, 3 = Northern. Ethiopia: 1 = Amhara, 2 = Oromia. Malawi: 1 = Northern, 2 = Central, 3 = Southern

**Table 5:** Cross-tabulation: gender and rural/urban location (Ghana and Malawi, weighted column percentages).

Gender	Ghana (N = 598)		Malawi (N = 580)	
	Urban % (SE)	Rural % (SE)	Urban % (SE)	Rural % (SE)
Female	83.2 (3.6)	69.8^**^ (4.4)	77.5 (4.1)	31.9^***^ (2.9)
Male	16.8 (3.6)	30.2 (4.4)	22.5 (4.1)	68.1 (2.9)

*Not:* Survey Chi2 analysis: rural–urban differences within each country: * p < 0.05, ** p < 0.01, ***p < 0.001.

Within both Ghana and Malawi, gender interacted with rural/urban location, with female CHWs disproportionately likely to be found in urban communities compared with males: Table 5. This effect was most pronounced in Malawi, with woman constituting over three-quarters (77.5%) of HSAs in urban areas and less than a third (31.9%) in rural ones. In Ghana, the majority of CHNs in both rural and urban areas were women, but the proportion of men in rural locations (30.2%) was nonetheless almost twice that in urban sites (16.8%). Our qualitative findings suggest that, in Malawi and Ethiopia, security concerns about women (especially younger women) living alone in isolated rural areas, and an expectation that married women should be co-located with their husbands, might act as strong deterrents for female CHWs to take up positions serving rural communities^17^ (see Steege, Taegtmeyer, et al., 2018, who noted similar concerns in other African countries).

### 5.2 Communications infrastructure and the reach of ‘formal’ mhealth

The survey data confirm the still limited reach of formal telecommunications and mhealth across the three countries: Table 6. Overall, fewer than 12% of CHWs reported having access to a functioning workplace landline, with rural clinics particularly poorly served, and only 15% had access to an ‘official’ mobile phone. Just under 17% were currently using any formal mhealth applications (either patient-facing applications or tools for data collection, reporting and logistics: Table 7).

**Table 6:** Workplace phone availability and the reach of formal mhealth, by country and by urban/rural locations within each country (weighted percentages). Base: whole sample.

	Ghana (N = 598)			Ethiopia (N = 1019)			Malawi (N = 580)			ALL (N = 2197)^c^
	Urban (N = 172)	Rural (N = 426)	All Ghana (N = 598)^a^	Urban (N = 143)	Rural (N = 876)	All Ethiopia (N = 1018)^b^	Urban (N = 148)	Rural (N = 432)	All Malawi (N = 580)	
Functioning Workplace landline										
Yes	**14.8%**	3.0%*	6.5%	**22.6%**	4.4%**	5.6%	**59.7%**	2.5%***	*26.0%^+++^*	11.7%
No	**85.2%**	**97.0%**	93.5%	**77.4%**	**95.6%**	94.4%	**40.3%**	**97.5%**	*74.0%*	88.2%

Ever received a mobile phone from workplace										
Yes, currently using	8.4%	19.4%	16.2%	0	5.4%	5.0%*	**15.4%**	32.5%**	*25.5%^+++^*	15.4%
Yes, but not currently	0	4.2%	2.9%	0	5.8%	5.4%	**6.1%**	**13.6%**	*10.5%*	6.11%
No, never	91.6%	76.4%	80.9%	100.0%	88.8%	89.6%	**78.5%**	**54.0%**	*64.0%*	78.1%

Ever used formal mhealth applications										
Yes, and still using	**4.3%**	12.2%*	*9.9%^+^*	4.4%	2.5%	2.6%	**22.5%**	49.5%***	*38.4%^+++^*	16.8%
Yes, but no longer use	**3.7%**	**6.1%**	*5.4%*	10.2%	22.3%	21.4%	**7.8%**	**17.5%**	*13.5%*	11.8%
No, never	**92.0%**	**81.7%**	*84.8%*	85.4%	75.3%	76.0%	**69.7%**	**33.0%**	*48.1%*	69.8%

Ever received solar charger or power bank from work										
Yes, still working	1.3%	3.1%	2.6%	1.1%	5.2%	4.9%	**5.5%**	14.6%*	*10.8%^+++^*	5.8%
Yes, but not working	0.5%	0.8%	0.8%	0.0%	2.8%	2.6%	**6.4%**	**16.0%**	*12.0%*	4.8%
No, never	98.1%	96.1%	96.7%	98.9%	92.0%	92.5%	**88.2%**	**69.4%**	*77.1%*	89.1%

*Notes:*

1. ^a^ denotes N = 597 for “Ever used mhealth applications”;

2. ^b^ denotes N = 1018 for “functioning landline”

3. ^c^ denotes N = 2196 for “Ever used mhealth applications” and “functioning landline”

4. Meta-analysis: differences between countries: statistically significant results shown in *italics*: ^+^ p < 0.05, ^++^ p < 0.01, ^+++^p < 0.001.

5. Survey Chi2 analysis: rural–urban differences within each country: statistically significant results shown in **bold**: * p < 0.05, ** p < 0.01, ***p < 0.001.

**Table 7:** Formal mhealth applications reported to be currently being used by CHWs (raw numbers, unweighted).

	Ghana (N = 598)	Ethiopia (N = 1019)	Malawi (N = 580)	All (N = 2197)
Total using formal mhealth applications	61	43	262	366
Child health/vaccination	14	21	102	137
Maternal/new-born health	11	24	46	81
Family planning	17	21	64	102
Disease outbreak	7	14	23	44
Medicine stock management	2	10	206	218
Managing/uploading data	18	19	51	88
Sending/uploading reports	8	13	147	168
Other	26	12	53	91

*Not using mhealth applications*	*537*	*976*	*318*	*1831*

As expected, exposure to formal mhealth was greater in Malawi than in Ghana or Ethiopia, reflecting differences in donor investment and national policy priorities; however, even in Malawi, only a minority were currently using mhealth applications. The prime focus of mhealth provision, especially in Malawi, was in rural areas, in line with UHC exigencies. A small proportion of CHWs (again, mostly in rural Malawi) had also received solar chargers or power banks to support phone use. Overall, however, the vast majority of CHWs surveyed remained apparently untouched by the (formal) ‘mhealth revolution’.

In the qualitative fieldwork, the patchy and unsustained nature of formal mhealth provision became even more evident. While most CHWs had no access to ‘official’ telecommunications, several HSAs in southern Malawi had received no fewer than three smartphones from different donors for different projects; some were still operational but most were not. Study participants in all three countries expressed frustrations similar to those reported in the literature: unsustained funding, insufficient financial/infrastructural support, and equipment failure. These excerpts are illustrative:

*“[Mhealth] Projects by NGOs are only lasting for two years and then phase out. […] They don’t continue. When phones are provided, they are eventually taken away.”* (Malawi, urban, male HSA)

*“In our woreda*^18^*there is a pilot project to send data to the server but the cost of airtime to sync with the server is too high; it was promised but they have not given us [phone credit] cards, so some HEWs are refusing to send the data.”* (Ethiopia, rural HEW)

*“One day we went for training and they told us we will be given phones, but we waited for a long time and it didn’t come. We talked and talked and talked about it but up till now the phones didn’t come.”* (Ghana, rural, female CHN)

One official in Ghana was very frank in his assessment of the challenges:

*“I am not sure the [Government] will be rolling out a full mHealth package for CHPs [Community Health Posts] in Ghana. There is a basic checklist of about 15 points for every CHPS compound; currently, many compounds do not even meet these criteria. The basic logistics like motorbikes are not available. The nurses have to use their own money to buy fuel and some of these things. So I don’t see government bypassing these to roll out mhealth.”*

### 5.3 The reach and application of informal mhealth

In contrast to the patchiness of official mhealth coverage, almost every CHW surveyed owned a personal mobile phone (Table 8), with many (21% in Ghana, 25% in Malawi) owning more than one. Smart-phone ownership was more widespread in Ghana (90%) and Malawi (70%) than in Ethiopia (40%), where there was also marked urban–rural variation: Table 9. Notably, almost every CHW surveyed reported using their personal mobile phone(s) in their work, with around 90% in each country doing this on a daily basis (median of 60 min per day). This was the case even where CHWs had access to ‘official’ phones, which were generally restricted to specific functions. Frequency of work-related personal phone use did not appear to vary by country, gender, age or rural/urban location: Table 9.

**Table 8:** CHWs’ personal mobile phone ownership and work-related usage (weighted percentages).

	Ghana	Ethiopia	Malawi	ALL
*All respondents*	*N = 597*^a^	*N = 1019*	*N = 580*	*N = 2196*
CHWs’ personal mobile phone ownership				
Currently own a working mobile phone	99.2%	98.5%	97.5%	98.7%
Currently own a non-working phone	0.7%	0.6%	0.4%	0.6%
Previously owned a mobile phone	0.2%	0.3%	2.1%*	0.3%
Never owned mobile phone	0	0.6%	0	–

*Current mobile phone owners only*	*N = 592*	*N = 1004*	*N = 565*	*N = 2196*

Ownership of internet-enabled phone	91.4%	38.4%*	71.7%	69.1%

Frequency of phone use for work				
Every day/most days	91.2%	87.6%	88.4%	89.3%
At least once a week	8.2%	11.6%	11.6%	10.5%
At least once a month	0.6%	0.4%	0.0%	0.5
Rarely or never	0	0.5%	0	–

Reported time (minutes) spent per day on work-related phone use: median (inter-quartile range)	60 (15–120)	60 (25–120)	60 (52–180)	60(30–120)

*Notes:*

1. ^a^ One missing response from Ghana sample (hence N = 597 rather than N = 598).

2. Meta-analysis: differences between countries: statistically significant results shown in **bold**: * p < 0.05, ** p < 0.01, ***p < 0.001.

**Table 9:** Survey logistic regression analysis: (a) having an internet-enabled phone and (b) using a personal mobile phone for work ‘every day or most days’. Base: all mobile phone owners.

Country		(a) Ownership of internet-enabled phone	(b) Use of personal mobile phone for CHW work ‘every day or most days’
		Odds Ratio (95% CI)	Odds Ratio (95% CI)
Ghana (N = 592)	Gender		
	Male	Reference	Reference
	Female	1.01 (0.42, 2.43)	0.44 (0.16, 1.17)
	Age		
	Per additional year	0.99 (0.92, 1.08)	1.01 (0.94, 1.08)
	Location		
	Urban	Reference	Reference
	Rural	2.02 (1.20, 3.41)*	1.00 (0.39, 2.57)

Ethiopia (N = 1004)	Age		
	Per additional year	0.99 (0.96, 1.03)	0.98 (0.90, 1.07)
	Location		
	Urban	Reference	Reference
	Rural	0.10 (0.03, 0.29)***	1.16 (0.52, 2.57)

Malawi (N = 565)	Gender		
	Male	Reference	Reference
	Female	1.41 (0.81, 2.47)	0.77 (0.40, 1.51)
	Age		
	Per additional year	0.99 (0.96, 1.04)	1.02 (0.96, 1.09)
	Location		
	Urban	Reference	Reference
	Rural	0.43 (0.21, 0.91)*	0.62 (0.27, 1.42)

*Note:* Odds ratios are reported, with 95% confidence intervals in brackets. Statistically significant results shown in **bold**: * p < 0.05, ** p < 0.01, ***p < 0.001.

CHWs used their phones in many different ways in order to communicate with patients, volunteers and colleagues; to facilitate data collection and reporting; to seek information/advice and in many other aspects of their daily work: Table 10. As well as voice calls and SMS (text messages), group communication via WhatsApp (an internet-based mobile instant messaging (MIM) service), was popular among CHWs in Ghana and Malawi (but not in Ethiopia) for sharing information on meetings or training opportunities, coordinating work activities, and obtaining advice on difficult cases. Ghanaian CHWs made extensive use of internet searches and applications; other widely-used functions across all sites included: cameras, calculators, stopwatches and torches. The multi-utility of phones is well illustrated by this focus group exchange between CHNs in rural Ghana:

*“If you go to Google, you will find so many Apps that talk about diseases and their treatment. So, when you get a condition that you don’t know much about, you need to get that App to know how to treat it.”*

*“I use the calculator [for report writing]. The calculators we have are spoilt, sometimes no battery, so you to go to your phone.”*

*“When a woman comes to deliver and there is a tear, we normally will have to use the torchlight on our phones to look through the vagina in order to be able to suture.”*

**Table 10:** Reported ‘informal mhealth’ usage in the preceding 4 weeks (weighted percentages). Base: all current mobile phone users.

	Ghana (N = 592)	Ethiopia (N = 1004)	Malawi (N = 565)	ALL (N = 2161)
Direct communication (with clients, volunteers, colleagues, etc.)				
Voice calls	98.6%	100.0%	99.3%	99.0%
SMS/text messaging	72.3%	50.7%	95.4%	73.6%
WhatsApp (or similar) to contact an individual	62.9%	3.6%***	77.0%	47.8%
Work-related group chat (e.g. WhatsApp)	78.2%	1.2%***	75.5%	51.6%

Data collection and reporting				
Camera: capturing reports/paperwork	69.7%	21.5%***	64.9%	52.3%
Calculator: for collecting data or making reports	89.4%	74.6%*	94.9%	87.8%
Sending data/reports via SMS/text	41.6%	33.8%	75.8%***	50.8%
Sending data/reports via WhatsApp (or similar)	61.1%	7.2%***	70.6%	46.4%

Seeking information/functions online				
Via Facebook or other social media	18.5%	28.3%	16.2%	17.8%
Via Google or other internet search	74.2%***	12.9%	17.3%	34.9%
Via Playstore (or similar) for health-related Apps	35.0%***	2.0%	5.4%	13.9%

Other tools/functions				
Notepad (or similar) for making notes	8.7%	2.1%	16.4%*	8.9%
Camera/video to record activities/events	74.2%	24.0%***	69.9%	56.4%
Camera to take images of patient symptoms	56.7%***	17.0%	26.8%	33.5%
Voice recording (e.g. for recording meetings)	12.1%	16.5%	33.0%***	20.6%
Calculator: calculating medicine dosages	78.4%	59.7%*	88.6%	76.6%
Torch to work in the night	60.5%	62.9%	69.2%	65.7%
Torch for patient examination	35.6%	38.5%	30.0%	32.7%
Stopwatch (taking pulse or breathing rate)	36.9%	23.8%	45.0%	37.2%

*Note:* Meta-analysis: differences between countries: statistically significant results shown in **bold**: * p < 0.05, ** p < 0.01, ***p < 0.001.

Multivariate within-country analysis indicates some rural/urban differences in phone usage, especially in Ethiopia and Malawi, where rural CHWs reported more limited use of certain ‘higher-tech’ phone functions (e.g. internet and cameras), perhaps reflecting the lower smartphone penetration: Table 11. Torches, calculators and stop-watches were widely used across all sites, but it was in the qualitative work that the value of these ‘basic’ functions in rural areas became particularly clear, as the excerpts above illustrate. Gender differences are also apparent in Ghana and Malawi, with male CHWs more likely than their female colleagues to report using some more ‘basic’ phone functions; it is not clear why this might be the case other than perhaps as a residual effect of the higher proportions of male CHWs working in rural locations. In Malawi, use of some phone features was also more limited among older respondents.

**Table 11:** Survey logistic regression analysis: Reported informal mhealth usage in the last four weeks. Base: all current phone owners.

Country		SMS (text)	Internet usage	Camera	Calculator	Stopwatch	Torch
		OR (95% CI)	OR (95% CI)	OR (95% CI)	OR (95% CI)	OR (95% CI)	OR (95% CI)
Ghana (N = 592)	Gender						
	Male	Reference	Reference	Reference	Reference	Reference	Reference
	Female	0.80 (0.59, 1.09)	0.98 (0.41, 2.31)	0.64 (0.46, 0.89)*	0.95 (0.51, 1.80)	0.59 (0.36, 0.97)*	0.68 (0.44, 1.05)
	Age						
	Per additional year	1.02 (0.95, 1.10)	0.99 (0.94, 1.05)	1.02 (0.46, 1.08)	1.05 (0.97, 1.13)	1.02 (0.98, 1.06)	1.03 (0.99, 1.08)
	Location						
	Urban	Reference	Reference	Reference	Reference	Reference	Reference
	Rural	1.78 (1.18, 2.69)	2.02 (1.29, 3.18)	1.37 (0.80, 2.33)	1.06 (0.54, 2.05)	1.05 (0.53, 2.05)	2.00 (0.93, 4.26)

Ethiopia (N = 1004)	Age						
	Per additional year	0.97 (0.91, 1.03)	1.00 (0.95, 1.06)	1.03 (0.98, 1.08)	1.02 (0.98, 1.05)	1.04 (0.95, 1.14)	1.04 (0.97, 1.10)
	Location						
	Urban	Reference	Reference	Reference	Reference	Reference	Reference
	Rural	0.82 (0.18, 3.64)	0.18 (0.07, 0.44)***	0.31 (0.18, 0.52)***	1.18 (0.33, 4.23)	2.12 (0.50, 9.04)	0.59 (0.19, 1.83)

Malawi (N = 565)	Gender						
	Male	Reference	Reference	Reference	Reference	Reference	Reference
	Female	0.43 (0.14, 1.31)	1.27 (0.69, 2.34)	0.71 (0.43, 1.16)	0.80 (0.26, 2.50)	0.29 (0.18, 0.48)***	0.52 (0.30, 0.91)*
	Age						
	Per additional year	0.91 (0.84, 0.98)*	0.97 (0.93, 1.01)	0.96 (0.91, 1.00)*	0.88 (0.81, 0.97)**	0.96 (0.92, 0.99)*	0.98 (0.94, 1.02)
	Location						
	Urban	Reference	Reference	Reference	Reference	Reference	Reference
	Rural	1.23 (0.39, 3.96)	0.41 (0.17, 0.98)*	0.54 (0.27, 1.08)	1.52 (0.64, 3.63)	1.16 (0.73, 1.85)	1.35 (0.88, 2.08)

*Note:* Odds ratios are reported, with 95% confidence intervals in brackets. Statistically significant results are shown in **bold**: * p < 0.05, ** p < 0.01, ***p < 0.001.

### 5.4 Positive evaluations of informal mhealth

When asked to evaluate the impact of informal mobile phone use on their work, CHWs across all three countries were overwhelmingly positive, with relatively few differences between or within countries. Phones had reportedly helped to make work more manageable and had enhanced both the *quantity* and *quality* of communication, with direct (reported) effects on patient outcomes: Table 12. On the whole, older respondents were most likely to report benefits (although with variation between countries): Table 13, Table 14. Rural-urban differences were surprisingly few in the survey data, but emerged much more strongly in the qualitative work.

**Table 12:** Perceived benefits of using personal phones for CHW work. Base: CHWs reporting use of own mobile phone for work.

	Proportions of CHWs reporting benefit (weighted percentages)			
	Ghana (N = 580)	Ethiopia (N = 1004)	Malawi (N = 565)	ALL (N = 2149)
Facilitating work and logistics				
Reduction of workload	95.9%^a^	99.4%^b*^	97.8%	98.2%^c^
Making work more efficient	99.2%	95.8%	99.0%	99.1%
Completing work faster	98.4%	99.4%*	97.3%	98.5%
Less need to travel	98.0%	99.7%**	97.4%	98.6%
Timely submission of reports data	94.3%	99.7%	97.2%	97.2%
Easier to arrange meetings/other logistics	99.0%	99.7%	99.6%	99.6%
Makes work more enjoyable	96.2%	90.0%*	99.4%	95.6%
Makes work less stressful	96.0%	88.4%*	94.6%	93.9%
Improving medicine supply	93.8%	96.4%	93.9%	95.0%

Improved communication				
Better communication with patients/clients	95.9%	98.7%	81.7%***	92.2%
Better communication with volunteers	85.5%***	99.1%	99.4%	94.8%
Better communication with colleagues/boss	99.2%	99.8%	99.5%	99.6%
Improving patient confidentiality	89.0%	89.4%	90.1%	89.3%
Easier patient follow-up	96.7%	97.6%	97.7%	97.4%

Improved outcomes				
Reduces risk of patients defaulting on meds	95.5%	98.5%^**^	95.2%	96.5%
Improving patient health outcomes	97.3%	92.8%	98.1%	97.7%
Directly saved a patient’s life	77.1%^a***^	91.6%^b^	88.3%	85.8%^c^

*Notes:*

1. a denotes N = 592; b denotes N = 1006; c denotes N = 2163

2. Meta-analysis: differences between countries: statistically significant results shown in **bold**: * p < 0.05, ** p < 0.01, ***p < 0.001.

**Table 13:** Survey logistic regression analysis: reported informal mhealth benefits (a): facilitating work & logistics. Base: CHWs reporting use of personal mobile phone for work.

		(a) Reduction of workload^b^	(b) Making work more efficient	(c) Completing work faster	(d) Less need to travel	(e) Timely submission of reports data	(f) Easier to arrange meetings / other logistics	(g) Makes work more enjoyable	(h) Makes work less stressful	(i) Improving medicine supply
		OR (95% CI)	OR (95% CI)	OR (95% CI)	OR (95% CI)	OR (95% CI)	OR (95% CI)	OR (95% CI)	OR (95% CI)	OR (95% CI)
Ghana (N = 580)	Gender									
	Male	Reference	Reference	Reference	Reference	Reference	Reference	Reference	Reference	Reference
	Female	0.53 (0.16, 1.81)	0.62 (0.04, 8.93)	1^a^	0.86 (0.08, 8.80)	1.11 (0.48, 2.57)	1^a^	0.71 (0.29, 1.72)	0.28 (0.07, 1.08)	0.889 (0.50, 1.59)
	Age									
	Per additional yr	.04 (0.94, 1.45)	1.01 (0.86, 1.18)	1.17 (0.88, 1.55)	1.06 (0.95, 1.19)	1.15 (1.05, 1.27)**	1.23 (1.11, 1.37)***	1.10 (0.98, 1.23)	1.05 (0.96, 1.14)	1.10 (1.00, 1.20)
	Location									
	Urban	Reference	Reference	Reference	Reference	Reference	Reference	Reference	Reference	Reference
	Rural	1.02 (0.37, 2.79)	0.61 (0.05, 7.19)	0.96 (0.29, 3.21)	1.19 (0.39, 3.65)	3.88 (1.83, 8.21)**	1.92 (0.18, 20.65)	1.02 (0.27, 3.87)	1.44 (0.64, 3.30)	2.20 (0.93, 5.21)

Ethiopia (N = 1004)	Age									
	Per additional yr	1.10 (0.83, 1.45)	1.08 (1.00, 1.16)*	0.98 (0.85, 1.14)	1.104 (0.92, 1.32)	1.069 (0.93, 1.22)	0.952 (0.77, 1.18)	0.991 (0.91, 1.08)	1.037 (0.94, 1.14)	1.015 (0.90, 1.14)
	Location									
	Urban	Reference	Reference	Reference	Reference	Reference	Reference	Reference	Reference	Reference
	Rural	0.46 (0.03, 6.35)	0.16 (0.02, 1.75)	1^a^	1^a^	1.82 (0.09, 35.87)	1^a^	0.80 (0.36, 1.77)	0.85 (0.24, 3.04)	2.14 (0.63, 7.28)

Malawi (N = 565)	Gender									
	Male	Reference	Reference	Reference	Reference	Reference	Reference	Reference	Reference	Reference
	Female	0.86 (0.09, 8.36)	0.30 (0.03, 3.57)	0.58 (0.11, 3.08)	0.34 (0.05, 2.54)	0.96 (0.24, 3.79)	0.01 (0.00, 0.07)***	2.00 (0.68, 5.95)	0.35 (0.13, 0.91)*	0.68 (0.22, 2.14)
	Age									
	Per additional yr	0.96 (0.89, 1.04)	1.14 (0.97, 1.34)	1.02 (0.95, 1.10)	1.15 (1.04, 1.27)**	0.97 (0.89, 1.05)	1.05 (0.92, 1.19)	1.07 (0.90, 1.28)	0.93 (0.85, 1.03)	1.05 (0.93, 1.18)
	Location									
	Urban	Reference	Reference	Reference	Reference	Reference	Reference	Reference	Reference	Reference
	Rural	0.99 (0.11, 9.04)	0.38 (0.04, 3.64)	1.29 (0.31, 5.38)	0.89 (0.10, 7.61)	1.34 (0.35, 5.07)	1^a^	1.16 (0.14, 9.33)	0.78 (0.29, 2.10)	1.95 (0.66, 5.77)

*Notes*

1. Odds ratios are reported, with 95% confidence intervals in brackets. Statistically significant results shown in **bold**: * p < 0.05, ** p < 0.01, ***p < 0.001.

2. a Denotes omitted.

3. b Denotes N = 592 for Ghana and N = 1006 for Ethiopia.

**Table 14:** Survey logistic regression analysis: reported informal mhealth benefits (b): improved communication and patient outcomes. Base: CHWs reporting use of personal mobile phone for work.

		(j) Better communication with patients/ clients	(k) Better communication with volunteers	(l) Better communication with colleagues/boss	(m) Improving patient confidentiality	(n) Easier patient follow-up	(o) Reduces risk of patients defaulting on medication	(p) Improving patient health outcomes	(q) Directly saved a patient’s life^b^
		OR (95% CI)	OR (95% CI)	OR (95% CI)	OR (95% CI)	OR (95% CI)	OR (95% CI)	OR (95% CI)	OR (95% CI)
Ghana (N=580)	Gender								
	Male	Reference	Reference	Reference	Reference	Reference	Reference	Reference	Reference
	Female	1.82 (0.65, 5.14)	0.54 (0.28, 1.01)	1.10 (0.15, 8.33)	1.28 (0.52, 3.18)	1.39 (0.25, 7.63)	1.32 (0.37, 4.68)	1.48 (0.19, 11.70)	1.16 (0.77, 1.73)
	Age								
	Per additional year	1.16 (1.00, 1.35)	1.06 (1.02. 1.11)*	0.98 (0.82, 1.17)	1.11 (1.03, 1.19)*	1.24 (1.02, 1.49)	1.13 (1.00, 1.28)	1.22 (0.98, 1.52)	1.09 (1.00, 1.19)
	Location								
	Urban	Reference	Reference	Reference	Reference	Reference	Reference	Reference	Reference
	Rural	2.30 (0.64, 8.25)	5.52 (2.89, 10.55)***	3.05 (0.43, 21.58)	1.57 (0.80, 3.06)	1.88 (0.31, 11.27)	1.64 (0.48, 564)	1.79 (0.37, 8.73)	1.35 (0.80, 2.28)

Ethiopia (N=1004)	Age								
	Per additional year	0.94 (0.79, 1.12)	.15 (0.93, 1.42)	0.82 (0.61, 1.09)	0.97 (0.88, 1.06)	1.01 (0.92, 1.11)	0.88 (0.72, 1.07)	1.03 (0.96, 1.11)	1.08 (0.93, 1.25)
	Location								
	Urban	Reference	Reference	Reference	Reference	Reference	Reference	Reference	Reference
	Rural	0.39 (0.04, 4.23)	22.90 (5.23, 100.20)***	1^a^	0.70 (0.41, 1.18)	1.95 (0.46, 8.31)	0.24 (0.02, 3.47)	0.71 (0.19, 2.63)	1.75 (0.61, 5.01)

Malawi (N=565)	Gender								
	Male	Reference	Reference	Reference	Reference	Reference	Reference	Reference	Reference
	Female	0.59 (0.34, 1.01)	0.73 (0.01, 43.30)	1^a^	0.55 (0.26, 1.19)	2.57 (0.42, 15.56)	0.95 (0.34, 2.66)	0.62 (0.17, 2.30)	0.93(0.49, 1.76)
	Age								
	Per additional year	0.94 (0.91, 0.98)**	0.89 (0.79, 1.01)	0.89 (0.86, 0.91)***	0.97 (0.91, 1.03)	0.97 (0.88, 1.06)	0.95 (0.86, 1.04)	1.00 (0.91, 1.10)	1.04 (0.98, 1.11)
	Location								
	Urban	Reference	Reference	Reference	Reference	Reference	Reference	Reference	Reference
	Rural	0.51 (0.27, 0.97)*	0.85 (0.01, 48.53)	1^a^	1.28 (0.51, 3.20)	0.52 (0.08, 3.43)	1.19 (0.53, 2.67)	0.19 (0.03, 1.33)	1.49 (0.73, 3.05)

*Notes*

1. Odds ratios are reported, with 95% confidence intervals in brackets. Statistically significant results shown in **bold**. * p < 0.05, ** p < 0.01, ***p < 0.001

2. a Denotes omitted.

3. b Denotes N = 592 for Ghana and N = 1006 for Ethiopia.

#### 5.4.1 Facilitating workloads and logistics

In both the survey and the qualitative work, the vast majority of CHWs claimed that using phones had made their workloads more manageable, more enjoyable and less stressful. Interviewees talked enthusiastically about wasted journeys becoming a thing of the past (e.g. travelling to a distant village to find everyone had left for a funeral), saving both time and money. Through group chat, meetings could be arranged and re-arranged in real time as circumstances changed, and CHWs could obtain assistance from their peers. The account of this rural HSA in Malawi is illustrative:

*“The [WhatsApp] group helps us when we don’t have supplies at the health centre. We do not even have to wait for the in-charge or pharmacy personnel; we just post on the WhatsApp group. […] And when you are stuck with something, for example examining a child and you see a symptom you don’t recognise, you just snap it and post on the WhatsApp group, and get feedback right there.”*

Phone communication also facilitated logistics for patients; for example, it was now possible to call ahead to check a health-worker’s availability before setting out, or indeed to ascertain the need for in-person consultation:

*“Before mobile phones, you had to go to the health post but you only had 50:50 chance to find health extension workers there […] but now you can call and ask whether they are available; even we can check whether the different medicines are available before going.”* (Ethiopia, rural, young man)

*“When I give the medicine to my child and still I don’t see any improvement, I can call and [the CHN] will tell me what to do and whether I need to bring the child back.”* (Ghana, rural mother)

#### 5.4.2 Improved quality of communication

The vast majority of CHWs surveyed claimed that phones had improved, not just the *quantity* of communication (with patients, volunteers and colleagues), but also its *quality*, fostering greater understanding and trust. See, for example, this focus group exchange in northern Ghana:

*“[The phone] brings you into close contact with the community. For instance, if you are able to reach out to a client or the community through a volunteer about anything concerning their health, it shows them that you really care about them. […] you are able to build a good relationship.”*

*“It also ensures that there is some kind of bond between you and your fellow workers. If you come to work and you don’t see someone, you quickly can pick the phone to call and find out the reason. […] Anytime I am in the house and someone calls to check on me, it makes me feel that at least there is somebody thinking about my welfare.”*

Phones can also facilitate confidentiality, as this (urban-based) Malawian HSA explained:

*“If someone has a sexually transmitted disease, they wouldn’t feel comfortable to come to a clinic because they would feel exposed; it won’t be confidential. So they can call the HSA directly.”*

#### 5.4.3 Impacts on health outcomes

Overall, nearly 98% of CHWs surveyed stated that their use of a personal mobile phone had resulted directly in improved patient outcomes; 85% claimed that it had saved someone’s life. Many such cases were recounted in interviews and focus groups. For example, in a remote Ethiopian village, one interviewee recalled a young woman who began haemorrhaging badly during labour. “I called the HEW,” she explained, “and we took the women to the health centre with a *bajaj.*^19^ She was bleeding in the transport so her life was at risk.” In another case, in Malawi, an HSA described attending to a child in the night who was convulsing and showing signs of acute cerebral malaria. In her words, “I immediately called the doctor to come and attend to this patient.”

#### 5.4.4 From beneficial to indispensable

Such were their apparent benefits, that mobile phones were generally regarded as being not just *beneficial* for community health-work, but *essential*. 73% of CHWs surveyed in Ghana, 87% in Ethiopia and 64% in Malawi reckoned that their work would be ‘very difficult’ or ‘impossible’ without a working phone, even for a limited period. One (rural) Ghanaian CHN put it succinctly: “A health-worker without phone? I don’t think so! It is something you can’t live without. With our profession, it is a must.”

Indeed, in Ghana and Malawi, where internet-based communication is increasingly normalised, not having a smartphone can be challenging, as this rural CHN (Ghana) explained:

“*When I was first posted, I was using a ‘yam phone’*^20^*[basic phone]. But along the line, I was forced to get this [smart]phone, due to the pressure and demands of the work. […] Personally, I wouldn’t have gone in for the Android had it not been the nature of my work. I was very OK with my ‘yam’ especially due to the battery life.”*

### 5.5 Challenges and costs

Despite the overwhelmingly positive evaluations, our study participants also drew attention to various challenges associated with ‘informal mhealth’, including: practical/infrastructural constraints; financial costs; threats to personal wellbeing; and concerns about confidentiality: Table 15.

**Table 15:** Perceived costs and challenges of using personal phones for CHW work (weighted percentages). Base: CHWs reporting use of own mobile phone for work.

Perceived challenges/drawbacks of informal mhealth	Weighted percentages of CHWs (phone owners) reporting challenge			
	Ghana (N = 580)	Ethiopia (N = 1004)	Malawi (N = 565)	ALL (N = 2149)
Practical & infrastructural				
Difficulty getting phone signal	81.0%^a^	93.7%^b**^	75.5%	83.3%^c^
Difficulty getting internet	80.4%	83.8%	59.9%***	74.0%
Difficulty re battery charging	46.1%***	90.9%	87.7%	75.0%
Phone not working	47.8%*	87.1%	72.3%	68.9%
Phone getting lost/stolen	24.0%	82.5%*	47.2%	50.9%
Clients without phones	85.4%	93.3%*	80.0%	86.0%

Financial				
Cost of buying airtime	80.8%***	99.3%	95.4%	91.9%
Cost of buying data	77.3%	73.9%	73.1%	76.1%

Threats to personal wellbeing				
Disturbance to regular work	39.5%	71.1%***	34.0%	46.8%
Disturbance: after-hours calls	47.2%	69.4%	31.4%	47.9%
Disturbance: night calls	44.9%	72.3%*	26.2%	46.9%
Unwanted/irrelevant calls	42.6%	66.2%*	27.4%	43.7%
Effects on personal relationships	25.1%	62.1%***	20.2%	33.6%
Increased workload	28.3%	60.7%*	15.6%	32.7%
Increased work-related stress	25.5%	53.4%*	11.6%	28.8%

Patient care				
Risks to confidentiality/privacy	24.4%	61.2%**	23.6%	33.7%

*Notes:*

1. a denotes N = 592; b denotes N = 1006; c denotes N = 2163

2. Meta-analysis: differences between countries: statistically significant results shown in **bold**: * p < 0.05, ** p < 0.01, ***p < 0.001.

#### 5.5.1 Practical and infrastructural constraints

First, the daily challenges of maintaining working phones with poor network connectivity and unreliable electricity supplies were widely reported across all sites, but particularly in Ethiopia, where telecommunications infrastructure remains relatively limited: Table 15. In all three countries, rural CHWs were significantly more likely than their urban counterparts to report difficulties in getting a phone signal: Table 16. Rural CHWs were also more likely to report other practical/infrastructural challenges, including internet connectivity (Ghana) and battery charging (Ethiopia).

**Table 16:** Survey logistic regression analysis: Reported practical/infrastructural and financial challenges. Base: CHWs reporting use of personal mobile phone for work.

		(a) Difficulty getting phone signal ^a^	(b) Difficulty getting internet	(c) Difficulty with battery charging	(d) Cost of buying phone credit (airtime)	(e) Costs of buying data	(f) Phone getting broken	(g) Phone getting lost or stolen	(h) Patients or others not having phone
		OR (95% CI)	OR (95% CI)	OR (95% CI)	OR (95% CI)	OR (95% CI)	OR (95% CI)	OR (95% CI)	OR (95% CI)
Ghana (N = 580)	Gender								
	Male	Reference	Reference	Reference	Reference	Reference	Reference	Reference	Reference
	Female	0.66 (0.42, 1.05)	0.68 (0.39, 1.16)	0.70 (0.36, 1.37)	0.75 (0.48, 1.78)	0.85 (0.57, 1.27)	0.78 (0.47, 1.27)	0.77 (0.46, 1.30)	1.29 (0.82, 2.02)
	Age								
	Per additional year	1.00 (0.96, 1.03)	0.98 (0.95, 1.02)	0.97 (0.94, 1.01)	0.99 (0.96, 1.03)	0.97 (0.94, 1.00)	0.98 (0.94, 1.01)	1.03 (0.98, 1.09)	1.01 (0.92, 1.10)
	Location								
	Urban	Reference	Reference	Reference	Reference	Reference	Reference	Reference	Reference
	Rural	1.95 (1.14, 3.34)*	2.40 (1.45, 3.97)**	1.38 (0.83, 2.27)	0.60 (0.28, 1.28)	0.83 (0.49, 1.41)	0.90 (0.49, 1.65)	0.89 (0.46, 1.70)	0.91 (0.31, 2.67)

Ethiopia (N = 1004)	Age								
	Per additional year	0.87 (0.78, 0.98)*	0.91 (0.81, 1.02)	0.94 (0.81, 1.09)	0.75 (0.65, 0.87)***	0.91 (0.83, 1.00)	0.95 (0.90, 0.99)*	0.97 (0.92, 1.03)	0.95 (0.85, 1.06)
	Location								
	Urban	Reference	Reference	Reference	Reference	Reference	Reference	Reference	Reference
	Rural	3.48 (1.08, 11.21)*	1.59 (0.53, 4.79)	3.41 (1.27, 9.15)*	9.16 (0.67, 125.66)	0.95 (0.35, 2.60)	1.52 (0.55, 4.18)	1.62 (0.61, 4.31)	1.32 (0.41, 4.17)

Malawi (N = 565)	Gender								
	Male	Reference	Reference	Reference	Reference	Reference	Reference	Reference	Reference
	Female	0.64 (0.38, 1.10)	1.12 (0.70, 1.80)	0.86 (0.47, 1.58)	1.30 (0.50, 3.35)	0.95 (0.53, 1.69)	1.29 (0.83, 1.99)	1.14 (0.79, 1.65)	0.75 (0.40, 1.38)
	Age								
	Per additional year	1.00 (0.96, 1.05)	0.97 (0.94, 0.998)*	1.03 (0.98, 1.08)	1.08 (0.94, 1.23)	0.97 (0.93, 1.02)	0.96 (0.92, 1.00)	0.99 (0.95, 1.04)	0.96 (0.91, 1.01)
	Location								
	Urban	Reference	Reference	Reference	Reference	Reference	eference	Reference	Reference
	Rural	1.75 (1.02, 3.01)*	1.11 (0.66, 1.87)	0.81 (0.43, 1.53)	1.80 (0.54, 6.02)	0.69 (0.31, 1.54)	0.91 (0.61, 1.35)	0.76 (0.53, 1.08)	0.73 (0.37, 1.42)

*Notes*

1. Odds ratios are reported, with 95% confidence intervals in brackets. Statistically significant results shown in **bold**: * p < 0.05, ** p < 0.01, ***p < 0.001.

2. a Denotes N = 592 for Ghana and N = 1006 for Ethiopia.**.**

Rural-urban differences emerged even more strongly in the qualitative work. Many rural CHWs described multiple constraints, faced on a daily basis; for example:

*“Sometimes even buying the credit is a problem – you go to town and all the stores are closed.”* (Ghana)

*“In this community, for Vodafone, there’s no signal. There is one particular tree you can stand by. […] You have put the phone up [in the tree] and have it on loudspeaker.”* (Ghana)

*“The challenge is keeping the phone charged throughout the week even in the face of power cuts.”* (Malawi)

*“More than the cost of mobile cards, what makes difficult for us is battery life of the mobile. We don’t have electricity access for charging, and the battery becomes depleted within 24 h. It becomes difficult to communicate with clients and office staff.”* (Ethiopia)

As a consequence of such challenges, more than two-thirds of CHWs surveyed (81% in Ghana, 68% in Ethiopia and 80% in Malawi) reported occasions within the preceding 12 months when their phones had become inoperable. Usually, interruptions last a few hours, until a network is restored, a battery can be charged or credit purchased; sometimes, however, they could extend to several days, weeks or even months. Temporary phonelessness (even short-term) reportedly hampered CHWs’ work significantly, leading to wasted journeys, missed meetings or logistical mix-ups, with detrimental impacts on patient care.

Altogether, 57 CHWs surveyed (2.6%) said that their inability to use a mobile phone when needed had resulted directly in a patient’s death. Although a relatively infrequent occurrence, its prominence in discussions underlined how traumatic these cases had been. One HSA in Malawi, for example, talked of her distress when she had been unable to call for help when a pregnant woman began bleeding because her phone was out of charge. The result was a stillbirth and, very nearly, a dead mother. In another case in rural Ethiopia, an HEW tried to call the health centre for help when a woman ingested poison after an argument with her husband. The recipient’s phone was not receiving calls so they ended up having to carry the woman on a make-shift stretcher; she died along the way.

#### 5.5.2 Financial costs

Second, CHWs reported having to meet the full costs of phone use themselves. Reported median weekly phone expenditure for work-related purposes was equivalent to £1.42/week in Ghana, £1.75/week in Ethiopia, and £1.25/week in Malawi, with males in Malawi apparently spending more than their female colleagues: Table 17. For low-paid CHWs, these amounts represent a significant proportion of average monthly salaries: over 4% in Ghana, 7% in Ethiopia and 6% in Malawi. In Ethiopia, younger HEWs in particular reported difficulties in affording phone credit (Table 15).

**Table 17:** Survey linear regression analysis: Reported weekly expenditure on ‘informal’ work-related phone use. Base: all CHWs using personal phone for work.

		Log total weekly phone spend for work in GBP
		B (95% CI)
Ghana (N = 580)	Gender	
	Male	Reference
	Female	−0.07 (−0.21, 0.06)
	Age	
	Per additional year	0.01 (−0.01, 0.02)
	Location	
	Urban	Reference
	Rural	−0.05 (−0.024, 0.13)

Ethiopia (N = 1004)	Age	
	Per additional year	0.028 (0.01, 0.05)**
	Location	
	Urban	Reference
	Rural	−0.18 (−0.40, 0.05)

Malawi (N = 565)	Gender	
	Male	Reference
	Female	−**0.33 (**−**0.49,** −0.16)***
	Age	
	Per additional year	0.011 (−0.01, 0.03)
	Location	
	Urban	Reference
	Rural	0.100 (−0.08, 0.28)

*Notes:* Statistically significant results shown in **bold**: * p < 0.05, ** p < 0.01, ***p < 0.001.

To support work-related phone expenditure, many CHWs (71.7% in Ghana, 48.1% in Ethiopia and 95.0% in Malawi) reported having had to make ‘sacrifices’ within the preceding four weeks, sometimes skimping on food or other ‘essentials’:

*“The card price is expensive. But, since it is very hard to work without a phone, we are spending money from our salary to buy phone card. Sometimes we minimize our monthly food expenditure to buy airtime.”* (Ethiopia, urban HEW)

*“Sometimes it happens that there’s no relish [an important component of the daily meal] at home but you need to make several communications and you only have 200 Kwacha. You would force your wife to look for anything and use the 200 Kwacha to buy airtime. […] In offices, I believe that bosses [receive free] airtime even though they get huge sums on their pay-slips compared to us.” (Malawi, rural, male HSA)*

While the HSA above was not alone in comparing his situation unfavourably with the ‘bosses’, most accepted this expenditure as part of the job, emphasising the qualities of selflessness and sense of vocation that were required of CHWs. One Ghanaian CHN declared that “the love for the work” was more important than worrying about airtime costs, while another interviewee from Malawi talked about the imperative to “sacrifice because we have helpful hearts.” The stakes – potentially saving a life – are seen to be too high to ‘worry about the cost’, as this rural Ethiopian HEW explained:

*“Most HDAs [Health Development Army volunteers] do not have any payment, so most of the time we have to call them back. Even though it is an expense, we can save the life of mothers, new-borns and accident victims, so I cannot worry about the cost.”*

#### 5.5.3 Threats to personal wellbeing

Beyond practical and financial issues, CHWs in all three countries (but particularly in Ethiopia) reported serious concerns about the impacts of phone use on their workloads and personal lives: Table 15. Within Ethiopia, such concerns were particularly prominent among rural HEWs and younger respondents, who reported multiple negative impacts, including: disturbance during and after work, unwanted calls, impacts on personal relationships, increased workloads and associated stress; younger HSAs in Malawi were also disproportionately likely to cite stress as a consequence of work-related phone use: Table 18.

**Table 18:** Survey logistic regression analysis: Reported disturbance and threats to wellbeing. Base: all CHWs using personal phones for work.

		(a) Disturbance to regular work	(b) Disturbance: after-hours calls	(c) Disturbance: night calls	(d) Unwanted calls	(e) Effects on personal relationships	(f) Increased workload	(g) Increased stress
		OR (95% CI)	OR (95% CI)	OR (95% CI)	OR (95% CI)	OR (95% CI)	OR (95% CI)	OR (95% CI)
Ghana (N = 580)	Gender							
	Male	Reference	Reference	Reference	Reference	Reference	Reference	Reference
	Female	1.06 (0.71, 1.57)	0.95 (0.68, 1.32)	0.93 (0.55, 1.55)	1.39 (0.82, 2.35)	1.02 (0.62, 1.70)	1.15 (0.69, 1.90)	1.17 (0.76, 1.80)
	Age							
	Per additional year	1.00 (0.96, 1.04)	1.01 (0.98, 1.03)	1.02 (0.99, 1.04)	1.01 (0.98, 1.03)	1.02 (0.99, 1.05)	0.99 (0.96, 1.03)	1.00 (0.94, 1.06)
	Location							
	Urban	Reference	Reference	Reference	Reference	Reference	Reference	Reference
	Rural	0.78 (0.51, 1.18)	0.74 (0.50, 1.10)	0.94 (0.63, 1.40)	1.01 (0.55, 1.84)	1.18 (0.81, 1.73)	0.73 (0.45, 1.17)	0.73 (0.37, 1.42)

Ethiopia (N = 1004)	Age							
	Per additional year	0.96 (0.93, 0.99)*	0.94 (0.90, 0.98)**	0.96 (0.90, 1.01)	0.93 (0.88, 0.98)*	0.92 (0.88, 0.96)***	0.92 (0.86, 0.98)*	0.93 (0.87, 0.98)**
	Location							
	Urban	Reference	Reference	Reference	Reference	Reference	Reference	Reference
	Rural	1.47 (0.52, 4.15)	1.79 (0.63, 5.09)	2.77 (0.91, 8.46)*	2.15 (0.76, 6.11)	2.11 (0.73, 6.06)	2.19 (0.70, 6.86)	3.29 (1.42, 7.58)**

Malawi (N = 565)	Gender							
	Male	Reference	Reference	Reference	Reference	Reference	Reference	Reference
	Female	1.31 (0.75, 2.30)	1.06 (0.67, 1.69)	0.96 (0.55, 1.66)	0.94 (0.57, 1.55)	0.84 (0.49, 1.43)	0.83 (0.43, 1.59)	0.98 (0.51, 1.87)
	Age							
	Per additional year	1.01 (0.97, 1.04)	1.02 (0.99, 1.05)	1.04 (0.998, 1.08)	0.99 (0.95, 1.03)	0.98 (0.93, 1.03)	0.95 (0.90, 0.997)*	0.95 (0.90, 1.01)
	Location							
	Urban	Reference	Reference	Reference	Reference	Reference	Reference	Reference
	Rural	0.73 (0.42, 1.27)	0.91 (0.54, 1.54)	0.88 (0.52, 1.48)	1.69 (0.99, 2.91)	1.38 (0.67, 2.83)	0.90 (0.49, 1.65)	0.78 (0.39, 1.54)

*Note:* Odds ratios are reported, with 95% confidence intervals in brackets. Statistically significant results shown in **bold**: * p < 0.05, ** p < 0.01, ***p < 0.001.

These themes were further developed in the qualitative work, with several CHWs highlighting new time-management challenges and risks to patient care:

*“Sometimes, while I am doing vaccination or ANC investigation, the director may call to request some urgent report. If you have several clients waiting in a queue, they may become disappointed. Sometimes I ask the director to wait until I finish with clients, but he may say that it is very urgent.”* (Ethiopia, rural HEW)

Others talked about the blurring of work/home boundaries, making it difficult ever to be fully ‘off duty’. Around two-thirds of CHWs surveyed in each country reported receiving work-related calls outside working hours, often at night, at least once a week, with those serving rural areas disproportionately affected: Table 19, Table 20. In the qualitative work, CHWs across all sites talked about being “disturbed” by night calls – often seen as unnecessary – that could “spoil our sleep” (as one Ghanaian CHN put it) and, in some cases, complicate personal relationships^21^:

*“The phone has also increased our work. Maybe after closing from work, you want to go and sleep but someone will call […] so yeah, it’s a challenge, because you can go the whole day without resting.”* (Ghana, rural CHN, male)

*“Someone in my catchment area was calling frequently. […] She would call me for useless things, even in the middle of the night when I was sleeping.”* (Malawi, rural, female HSA)

*“Usually it is women who call but, one time, a man called me at night because his wife was going into labour. My husband was angry and accused me of having an affair with man because he called me in the night”* (Ethiopia, rural HEW)

**Table 19:** CHWs reporting receiving after-hours calls, night calls and missed calls (‘flashes’) (weighted percentages).

Proportions reporting receiving calls more than once a week on average	Ghana (N = 580)	Ethiopia (N = 1004)	Malawi (N = 565)	ALL
Missed calls (‘flashes’)	49.4%***	79.3%	81.6%	69.8%
Calls outside working hours^a^	64.4%	71.6%	59.7%	62.8%
Night calls^b^	47.5%	64.6%	28.5%*	43.9%

*Note:*

1. a Ethiopia n = 1005

2. b Ghana n = 471; Ethiopia n = 948; Malawi n = 547

3. Statistically significant results shown in **bold**: * p < 0.05, ** p < 0.01, ***p < 0.001.

**Table 20:** Survey logistic regression analysis: CHW reports of receiving flashes, after-hours calls and night calls at least once a week. Base: CHWs reporting use of personal mobile phone for work.

		(a) Missed calls / flashes	(b) After-hours calls	(c) Night calls^a^
		OR (95% CI)	OR (95% CI)	OR (95% CI)
Ghana (N = 592)	Gender			
	Male	Reference	Reference	Reference
	Female	0.51 (0.26, 1.00)*	0.78 (0.59, 1.01)	0.88 (0.57, 1.36)
	Age			
	Per additional year	1.04 (1.00, 1.08)*	1.04 (1.02, 1.08)**	1.06 (1.02, 1.09)**
	Location			
	Urban	Reference	Reference	Reference
	Rural	1.30 (0.83, 2.04)	1.54 (1.21, 1.97)**	1.95 (1.21, 3.16)*

Ethiopia (N = 1004)	Age			
	Per additional year	0.95 (0.85, 1.07)	1.00 (0.94, 1.07)	1.03 (0.96, 1.11)
	Location			
	Urban	Reference	Reference	Reference
	Rural	0.45 (0.11, 1.83)	3.99 (0.84, 18.86)	5.96 (1.23, 28.97)*

Malawi (N = 565)	Gender			
	Male	Reference	Reference	Reference
	Female	0.80 (0.46, 1.37)	0.50 (0.33, 0.75)**	0.40 (0.22, 0.72)**
	Age			
	Per additional year	0.97 (0.92, 1.03)	1.01 (0.97, 1.05)	1.01 (0.97, 1.05)
	Location			
	Urban	Reference	Reference	Reference
	Rural	1.40 (0.76, 2.55)	1.50 (0.89, 2.54)	1.80 (0.99, 3.25)*

*Notes:*

1 ^a^ denotes N=580 in Ghana.

2. Odds ratios are reported, with 95% confidence intervals in brackets. Statistically significant results shown in **bold**: * p < 0.05, ** p < 0.01, ***p < 0.001.

CHWs also have to contend with widespread practice of ‘flashing’ or ‘beeping’: leaving intentionally missed calls for the receiver to return at their own cost. Usual etiquette demands that lower-status or poorer individuals (and sometimes women) flash higher-status or wealthier ones (and men; see Archambault, 2013, Archambault, 2017, on the gender dimensions of this practice). Although CHWs are underpaid compared with other health-workers, they are nonetheless perceived to be better-off and have higher social standing than other community members. Nearly half of CHWs surveyed in Ghana and four-fifths of those in Ethiopia and Malawi reported receiving work-related ‘flashes’ at least once a week: Table 19. No clear rural/urban differences were observed, but men in Ghana were reportedly more frequent recipients than women: Table 20. Missed calls can bring both financial and moral pressures. Returning every call immediately would be prohibitively expensive; however, *not* returning a missed call in an emergency situation could have disastrous consequences, as our study participants explained:

*“If you have airtime you ought to just call and find out why the person was calling, because if you avoid it, one day you will avoid news that could have saved someone’s life.”* (Malawi, rural, female HSA)

*“Some missed calls are very risky; for example from a labouring woman who is bleeding or a life-threatening accident. If I didn’t call back, the result can be very bad. Therefore we have to be always alert like a fire-fighter.”* (Ethiopia, rural HEW)

#### 5.5.4 Confidentiality

Finally, some study participants raised concerns around privacy and confidentiality. In each country, 20–25% of CHWs reported having unencrypted confidential information stored on their phones, from images of patient symptoms posted to a WhatsApp group for advice, to SMS messages communicating test results. This is a particular concern when phones are shared – a common practice throughout Africa (Porter et al., 2016, Porter et al., 2020). 67% of CHWs surveyed in Ghana, 25% in Ethiopia and 41% in Malawi reported regularly sharing their phones with colleagues, friends, family members, and sometimes patients; once the phone is ‘unlocked’, this information is visible to any user. Moreover, images uploaded to social media can continue to circulate through the internet after being deleted from individual phones; a source of concern for several study participants.

## 6 Discussion

### 6.1 Informal mhealth at scale in Africa

Let us return to the four original research questions. What is the reach of formal and informal mhealth? What forms does informal mhealth take? What are its perceived impacts? And how equitably are these distributed?

First, our data show unequivocally that ‘informal mhealth’ is happening *at scale* across Ghana, Malawi and Ethiopia, far out-stripping the reach of its ‘formal’ equivalent, despite more than a decade of planning and investment. Even in Malawi, which has enjoyed notable success stories, formal mhealth technologies had reached a minority of our study participants, while projects were often patchy and unsustained. In Ghana, and especially in Ethiopia, with its vast rural population and ongoing political instability, formal mhealth coverage remains minimal, with limited prospects for scale-up in the foreseeable future. By contrast, in all three countries, almost every CHW surveyed owned a personal mobile phone that they were using regularly and proactively in their work.

Addressing the second question, CHWs were using personal mobile phones in a variety of imaginative ways to address gaps in healthcare infrastructure and resourcing. The applications often mirrored – and sometimes went beyond – those promoted in formal mhealth programmes, even where people had no experience of those; for example: facilitating regular patient communication; managing medicine supplies; compiling/reporting data; seeking advice on case management; liaising with volunteers; and responding to emergency situations. As well as basic calling and texting, CHWs were using multiple phone functions to achieve these objectives, from internet platforms and cameras, to calculators, stopwatches and torches.

Thirdly, regarding (perceived) impacts, study participants were unequivocally positive in their evaluations. CHWs reported overwhelmingly that phones had helped them to manage demanding and unpredictable workloads, and had enhanced the *quality* of communication, with positive impacts on health outcomes. Patients also talked enthusiastically about the difference that phones had made to their interactions with CHWs.

However, our study participants also talked about the costs and challenges associated with informal mhealth, including those reported in previous qualitative studies (Mechael, 2009, Oliver, Geniets, Winters, Rega, & Mbae, 2015, Hampshire et al., 2017, Watkins et al., 2018). CHWs across all sites reported difficulties in keeping phones operational where power outages and network unreliability were a daily reality. Phones also brought financial burdens and other threats to personal wellbeing through the blurring of work/life boundaries and expectations to be constantly available; security of personal data was also a concern for some. As others have noted, weak regulation and enforcement can make digital health particularly vulnerable to security breaches in some LMICs (Bloom et al., 2017, Duclos et al., 2017, Hackett et al., 2018, Wallis et al., 2017). Other potential concerns, not mentioned by our respondents, include the risk that CHWs obtain information from unreliable internet sources, compromising patient care (see Buijink et al., 2013, Ebeling, 2011 on the commercial interests underpinning many ‘health information’ sites).

These findings give cause for concern, especially given that CHWs are typically underpaid and have high workloads (especially in this era of task shifting: see Smith et al., 2014, Zachariah et al., 2009, World Health Organization, 2008. Those in our study repeatedly said that they were willing to bear the costs of phone use because of their ‘helpful hearts’ and ‘love for the work’. Discourses around the selflessness and willingness to make personal sacrifices, can make CHWs especially vulnerable to exploitation (see also Hampshire et al., 2017; Brown and Green, 2015, Maes, 2014, Maes and Kalofonos, 2013, Nading, 2013, Prince, 2012). The possibility that informal mhealth might increase these vulnerabilities should not be taken lightly (Hampshire et al., 2017; Maes et al., 2011, Maes et al., 2019, Jenkins, 2009).

Moreover – and in answer to the final question – the benefits and costs may be unevenly, and inequitably, distributed between and within countries. Taken as a whole, our data do not suggest an *absolute* disadvantage to some groups from the informal digitalisation of healthcare, as Haenssgen (2018) identified in India and China. But they do point to a potential exacerbation of existing inequalities, especially between rural and urban areas. As noted above, the transformational potential of (formal) mhealth is widely held to be greatest in rural settings with limited physical infrastructure (World Health Organization, 2019). The same may be true for *informal* mhealth: in the absence of basic equipment and transport, ‘simple’ phone functions (like voice calls or a torch) could be live-saving in rural areas. However, the costs of informal mhealth may also be borne disproportionately by CHWs serving rural communities. This was apparent both in some survey responses and in the qualitative work, with rural CHWs telling distressing stories about being unable to get a phone signal, re-charge a battery, or purchase phone credit at a crucial moment, sometimes with devastating consequences.

Given the widely-reported gender dimensions of both the digital divide (Jennings and Gagliardi, 2013, World Bank, 2016) and health inequalities (e.g. Standing, 1997), it is perhaps surprising that gender differences did not emerge as more a significant factor in our study. There little indication from either the survey or the qualitative work that female CHWs were systematically disadvantaged in the adoption of these new practices. If anything, the opposite was true, especially in Malawi, where male HSAs reportedly spent more money on work-related calls and received more after-hours and night calls than their female colleagues; the same was true of males CHNs in Ghana receiving deliberately missed calls (‘flashes’). It is not clear from our data to what extent these differences are ‘real’ or an artefact of the gender distribution of CHWs across rural and urban areas.

However, within-country gender differences are only part of the story. The vast majority of CHWs worldwide are women. Globally, women make up around 70% of the healthcare workforce, but are concentrated in low-paid positions like CHWs, and unpaid voluntary roles, right at the bottom of professional hierarchies (Boniol et al., 2019, International Labour Organization, 2017, Langer et al., 2015). If CHWs are shouldering the burden of informal mhealth costs, it is likely that, at a global level, this is falling disproportionately on women. The risk that informal mhealth could further entrench the feminisation of under-remunerated care work (see also Molyneux, 2002, Jenkins, 2011, Brown, 2013, Maes and Kalofonos, 2013, Swartz, 2013, Steege et al., 2018) is compounded by the fact that the costs go unnoticed because they are borne privately by individuals (women) – a phenomenon noted by George (2008:75) over a decade ago:

*“[T]hese mainly female frontline health workers compensate for the shortcomings of health systems through individual adjustments, at times to the detriment of their own health and livelihoods. So long as these shortcomings remain as private, individual concerns of women, rather than the collective responsibility of gender, requiring public acknowledgement and resolution, health systems will continue to function in a skewed manner, serving to replicate inequalities in the health labour force and in society more broadly.”*

Age also plays a part. In general, across the three countries, older CHWs were more likely than their younger colleagues to report benefits of phone use (although the latter may have had limited experience of work *before* mobile phones). But it appears that younger CHWs may also bear the brunt of the costs; this was particularly evident in Ethiopia, where financial challenges, as well as concerns about unwanted calls, after-hours calls, and detrimental impacts on wellbeing, were disproportionately reported by younger HEWs. This may be partly a function of deeply-rooted anxieties about the (physical and moral) security of young women living alone in isolated rural areas noted above. The combined effects of gender, age and location in such settings may thus produce intersectional forms of disadvantage greater than the sum of their parts (see Larson, George, Morgan, & Poteat, 2016).

### 6.2 Interpretation and policy implications

These findings suggest further pressing questions: why has *informal* mhealth been so much more ‘successful’ than its formal equivalent? And how should policy-makers respond?

The first question appears more straightforward. Informal mhealth has happened spontaneously, from the bottom up, because it because it fulfils an important need on the ground. Unlike formal mhealth programmes, with their (usually) long lead-in times, informal mhealth is inherently flexible and responsive to new technologies, opportunities and the specificities of local situations. And, of course, it is cost-free from the perspective of governments and donors.

The second question is trickier: how should policy-makers respond when informal mhealth appears to have thrived not just *despite*, but *because of*, a lack of external intervention? One option is to do nothing and let informal mhealth continue to expand and fill infrastructural gaps. But, as we have seen, informal mhealth is not cost-free; the costs are inequitably borne by the lowest-paid health-workers (predominantly women at a global level), especially those serving rural areas. To do nothing means accepting, and perhaps reinforcing, existing inequities. On the other hand, is intervention of any kind possible without undermining the very basis of informal mhealth and creating yet another failed formal programme? This is not an easy circle to square, but we offer some tentative suggestions, all of which require CHWs to be right at the heart of decision-making processes.

First, we should never assume a *tabula rasa*^22^. Informal mhealth cannot do everything; some complex applications need expert planning and development. However, too many interventions are conceived in distant offices with little appreciation of *what is already happening on the ground*. Given that the vast majority of CHWs worldwide almost certainly own a mobile phone, it is imperative to start by finding out how these are currently being used. This approach has the dual advantage of avoiding replication (e.g. if there is already a functioning WhatsApp group, there may be no need to introduce a new platform) and capitalising on systems developed and tested ‘on the ground’. As one rural Ghanaian CHW put it:

*“One thing I would like to say is that, if they [NGO] are coming to initiate something, hardly do they come to the ground to find out. Some of them […] don’t know what we are going through here. They should come and experience real things on the ground for them to see before they start bringing new [projects] for us to just suffer and to put our frustrations on the clients.”*

Second, scalability need not mean uniformity. Across the three countries in our study, informal mhealth has taken different forms. For example, WhatsApp has become the medium of choice for CHW group communication across much of Ghana and Malawi, where internet-enabled phones are widespread and connectivity reasonably good while, in Ethiopia, voice calls remain the norm. Likewise, rates of literacy (including digital literacy) among the general population will in part determine the most appropriate communication platforms. Any national mhealth scale-up must therefore be responsive to difference, prioritising local experience and specificity over standardisation.

Third, we need to find ways to *build on* informal practices, without either ignoring or compromising them. In some cases, this could be achieved with relatively low-cost interventions; for example, providing support for CHWs to use their current phones rather than handing out new ones. When asked about possible ‘ways forward’, by far the most popular suggestion from focus group participants was some financial support to offset phone-related expenditure: either through direct credit transfers or by small supplements to monthly salaries. The latter is more in keeping with the spirit of informal mhealth, and was generally preferred as it would enable CHWs to choose networks with good local coverage and to take advantage of current ‘deals’; it would also allow for expenditure on phone charging and other maintenance costs. Study participants generally agreed that relatively modest sums (equivalent of GBP 2–3 per month) could make a difference for those struggling with hardship; others talked about the symbolic value in recognising CHWs’ ‘hidden sacrifice’. Others suggested developing guidelines/training on phone use, for both CHWs and community members, on topics such as managing expectations around availability, storage of confidential data, and assessing the reliability of online information. As with the other measures, to be effective, these must be developed *with and by* CHWs.

## 7 Conclusion

This study is the first, to our knowledge, to report on the health-workers’ ‘informal mhealth’ practices on a large scale. Our findings corroborate previous qualitative studies (Mechael, 2009; Oliver, Geniets, Winters, Rega, & Mbae, 2015; Hampshire et al., 2017; Mars and Scott, 2017, Watkins et al., 2018, Williams and Kovarik, 2018, Ling et al., 2020) in documenting the many creative ways that CHWs use personal mobile phones, with these practices becoming deeply interwoven into the fabric of *de facto* healthcare delivery. However, unlike those other studies, we have been able to show that informal mhealth is not an isolated or exceptional phenomenon; in contrast to the still limited reach of *formal* mhealth, it is already happening *at scale*.

Our study had some limitations. First, variation in sampling schemes across the three countries hinders direct comparison. Second, in a cross-sectional study based on self-reporting, we cannot make definitive inferences about patient outcomes. Likewise, estimates of time, money, numbers of calls, etc. are subject to recall errors and desirability bias, as discussed above. An important next step would be to conduct a prospective study, including objectively measurable outcomes. Third, while the qualitative data suggest the possibility of detrimental intersectional effects between gender, age and location, our surveyed was not powered sufficiently to analyse these interactions statistically. This would be another useful next step, requiring larger sample sizes.

These caveats notwithstanding, it is clear from our work that informal mhealth is a an important phenomenon: a *large-scale emergent health system* that deserves more systematic attention from researchers and policy-makers. We would encourage others to extend this research to other countries and contexts, and to broaden the scope of enquiry to include other healthcare professionals as well as non-medical staff (such as drivers, cleaners and security personnel). The resulting evidence base will be crucial in enabling governments and non-governmental actors others to work effectively in collaboration *with*, rather than *against*, existing practice.

## CRediT authorship contribution statement

**Kate Hampshire:** Conceptualization, Methodology, Formal analysis, Investigation, Writing - original draft, Supervision, Project administration, Funding acquisition. **Tawonga Mwase-Vuma:** Methodology, Investigation, Writing - review & editing. **Kassahun Alemu:** Methodology, Investigation, Writing - review & editing, Supervision, Project administration. **Abane Albert:** Writing - review & editing, Supervision, Project administration. **Alister Munthali:** Methodology, Writing - review & editing, Supervision, Project administration. **Tadesse Awoke:** Methodology, Investigation, Writing - review & editing. **Simon Mariwah:** Methodology, Investigation, Writing - review & editing. **Elita Chamdimba:** Methodology, Investigation, Writing - review & editing. **Samuel Asiedu Owusu:** Methodology, Investigation, Writing - review & editing. **Elsbeth Robson:** Methodology, Investigation, Writing - review & editing. **Michele Castelli:** Supervision, Investigation, Writing - review & editing. **Ziv Shkedy:** Methodology, Validation, Writing - review & editing. **Nicholas Shawa:** Software, Supervision, Writing - review & editing. **Jane Abel:** Software, Project administration, Writing - review & editing. **Adetayo Kasim:** Conceptualization, Software, Methodology, Writing - review & editing.

## Declaration of Competing Interest

The authors declare that they have no known competing financial interests or personal relationships that could have appeared to influence the work reported in this paper.

## Footnotes

1 Mhealth (mobile health) is defined by the WHO (2008: 6) as “medical and public health practice supported by mobile devices” including (but not limited to) mobile phones. It comes under the broader umbrella of eHealth (electronic health) and telemedicine (delivering healthcare at a distance using information and communication technologies). This paper focuses specifically on use of mobile phones in healthcare.

2 Debate about appropriate nomenclature (‘patients’, ‘clients’, ‘service users’) for people who access healthcare continues (Costa, Mercieca-Bebber, Tesson, Seidler, & Lopez, 2019). Here, we use these terms interchangeably.

3 We were not able to collect sufficiently reliable data on socio-economic status to enable analysis by income, the other widely-recognised correlate of digital exclusion.

4 GBP equivalents are based on approximate exchange rates at the time of fieldwork. Salary estimates are based on reported data from the survey; see below.

5 For more information on CHWs in the three countries, see: Baatiema, Sumah, Tang, and Ganle (2016) and Ghana Ministry of Health (2014) for Ghana; APC (2014), Smith et al. (2014) and Malawi Ministry of Health (2012) for Malawi; and Wang, Roman, Gandham, and Chala (2016) and Riegera et al., 2019 for (Ethiopia).

6 The project was funded by funded under the Health Systems Research Initiative, a partnership between UK Medical Research Council, UK Economic and Social Research Council, UK Aid and Wellcome Trust (MR/R003963/1).

7 The 28th District, the island of Likoma in Lake Malawi with a population of c. 14,500 (0.05% of the total population), was excluded for logistical reasons.

8 At the time when fieldwork began, Ghana was divided into 10 Regions. During fieldwork, following a national referendum, six new regions were created. Two of our study regions were affected: Brong Ahafo was divided into three new regions (Bono, Ahafo and Bono East) as was Northern Region (divided into Northern, Savannah and Northeast). Throughout this paper, we refer to the original Regions which were used for sampling.

9 CHAG (Christian Health Association of Ghana) and CHAM (Christian Health Association of Malawi) manage a substantial proportion of health facilities with publicly-funded CHWs. They are the second biggest healthcare providers in each country.

10 Different sites were used for piloting to those in the main survey.

11 For example, in the ‘impacts’ section, survey participants were presented with a list of possible benefits of informal mhealth and were asked to specify for each whether or not they personally had experienced this benefit; a similar procedure was used for possible challenges and costs. In each case, interviewers then prompted for additional benefits/challenges, etc. not covered by the pre-coded response categories, which were then post-hoc coded. In practice, there were very few of these, suggesting that our initial qualitative work and piloting had been successful in generating comprehensive lists of possible responses.

12 The sample size calculation was: n= ${\mathrm{FPC}}^{-1}\times z^{2}\times p\left(1-p\right)/e^{2}$, where FPC is a finite population correction factor defined as $1+{(z}_{\left(1-\alpha \right)}^{2}\times p\left(1-p\right))/Ne^{2}.$N denotes population size. z denotes z-score corresponding to 95% confidence interval for a Gaussian distribution. p denotes the expected proportion of people with mobile phone and we have assumed 50%. e denotes margin of margin of error. The required sample size using this formula was 384 for Ghana and Malawi and 786 for Ethiopia. However, assuming design effects of 25%, the minimum sample size required was 480 participants for Ghana and Malawi and 960 for Ethiopia.

13 18 respondents in Ghana and 2 in Ethiopia had to leave before the end of the interview and so did not respond to all the questions, resulting in some missing data; these are indicated as appropriate in the analyses. No replacements were used for non-responses.

14 For example, all CHWs in Ethiopia are women; the median age of CHWs in Malawi is ≥10 years greater than in the other two countries.

15 For this reason, those meta-analyses are not included in the paper but are available on request.

16 Many older interviewees, especially in Malawi and Ethiopia, preferred to give verbal consent.

17 Although CHWs are supposed come from, and live within, the communities they serve, this is often not the case. According to our survey data, fewer than half of CHWs surveyed in Ethiopia (48.8%) or Malawi (44.8%) lived within their catchment areas, compared with 81.8% in Ghana.

18 District in Ethiopia.

19 3-wheeler motorised vehicle widely used as taxis in Ethiopia (also known as auto-rickshaw or tuk-tuks).

20 So called because of a well-known advert in which having a basic phone is compared to giving yams for dowry instead of cattle.

21 Similarly in Tanzania, Feldhaus et al. (2015) found that both male and female CHWs were suspected of having ulterior, adulterous, motives for making home visits, causing family friction.

22 This is not a new observation; see, for example, Steven Polgar (1962:165) on ‘the fallacy of the empty vessel’.
