# Supplementary material for: Informal mhealth at scale in Africa: Opportunities and challenges
Source: World Dev. 2021 Apr;140:105257. doi: 10.1016/j.worlddev.2020.105257 (PMC7903241; doi:10.1016/j.worlddev.2020.105257)
Supplement: Supplementary Data 1 [file mmc1.pdf]

INFORMAL MHEALTH AT SCALE IN AFRICA: OPPORTUNITIES AND CHALLENGES

CONFLICTS OF INTEREST STATEMENT

None of the authors has any conflicts of interest.

*Credit Author Statement

INFORMAL MHEALTH AT SCALE IN AFRICA: OPPORTUNITIES AND CHALLENGES

AUTHOR CONTRIBUTIONS

Kate Hampshire: conceptualisation, methodology, formal analysis, investigation, writing – original draft, supervision, project administration, funding acquisition

Alister Munthali: methodology, writing – review & editing, supervision, project administration

Kassahun Alemu: methodology, investigation, writing – review & editing, supervision, project administration

Abane Albert: writing – review & editing, supervision, project administration

Elsbeth Robson: methodology, investigation, writing – review & editing

Simon Mariwah: methodology, investigation, writing – review & editing

Elita Chamdimba: methodology, investigation, writing – review & editing

Tawonga Mwase-Vuma: methodology, investigation, writing – review & editing

Tadesse Awoke: methodology, investigation, writing – review & editing

Samuel Asiedu Owusu: methodology, investigation, writing – review & editing

Michele Castelli: supervision, investigation, writing – review & editing

Ziv Shkedy: methodology, validation, writing – review & editing

Nicholas Shawa: software, supervision, writing – review & editing

Jane Abel: software, project administration, writing – review & editing

Adetayo Kasim: conceptualisation, supervision, methodology, writing – review & editing

IMAGINE (Informal Mhealth in Africa: Grassroots Initiatives & Networks)

QUESTIONNAIRE FOR COMMUNITY HEALTH WORKERS [Ethiopia: Health Extension Workers]

Preamble

(A) Questionnaire number [ ]

(B) Date: DD / MM / YY:______/_____/_____

(C) Time: HH/MM:______/______

(D) Interviewer ID: [___]

(E) Country: Ethiopia

(F) Region/State:
- Amhara [ ]
- Oromia [ ]

(G) Zone: ___________________________

(H) District (or sub-city): _________________________

(I) Location: [urban / rural]

(J) Name of Health Post where the HEW is based: _______________________________________

(K) Name of Cluster Health Centre: ____

(L) Does the Health Post have a landline or wireless telephone? If so, is it currently working?
- Yes – there is a landline/wireless phone which is currently working [ ]
- Yes - There is a landline/wireless phone but it is not currently working [ ]
- No – there is no landline/wireless phone [ ]

(M) Does the Cluster Health Centre have a landline or wireless telephone? If so, is it currently working?
- Yes – there is a landline/wireless phone which is currently working [ ]
- Yes - There is a landline/wireless phone but it is not currently working [ ]
- No – there is no landline/wireless phone [ ]

Go through introduction with informed consent procedure [separate document]

Section A: Background information on respondent

1. Gender [F / M] *[Tick one]*

2. Age [in completed years] ____________

3. Job title: [*Tick one*]
    Health Extension Worker [ ]

4. How much do you earn each month as an HEW…
    (a) Monthly salary (average) ________ Birr
    (b) Additional allowances, etc. (average) ________ Birr

5. How far is your Health Post from the Cluster Health Centre?
    (a) In kilometres: _______ km
    (b) In time to walk there: _______ hours [include fractions of an hour]

6. How far is your Health Post from the furthest part of your catchment area?
    (a) In kilometres: _______ km
    (b) In time to walk there: _______ hours [include fractions of an hour]

7. Do you live within your catchment area? [*Tick one*]
    Yes, all of the time [ ]
    Yes, some of the time [ ]
    No [ ]

8. Do you currently have any transport provided by your workplace to move around? [*Tick all that apply*]
    Yes – bicycle [ ]
    Yes – motorbike [ ]
    Yes – taxi/bajaj fares [ ]
    Yes – other [ ] → please state _________________________________
    No – none [ ]

9. Duration of employment as a HEW: [______] <u>months / years</u>

10. What is your level of training and qualification?

    (a) HEW training level: 1 / 2 / 3 / 4 [*Circle one*]

    (b) Additional nursing or midwifery qualifications: [*Tick all that apply*]
        Nursing Diploma [ ]
        Nursing Degree (BSc) [ ]
        Midwifery Diploma [ ]
        Midwifery Degree (BSc) [ ]
        Other [ ] → specify _______________________________
        None [ ]

Section B: Phone (and tablet) ownership and use

11. Have you ever received a mobile phone or tablet **from your work** (Ministry, donors, etc.)? Do you currently have one? Is it working? *[Tick one]*

    I currently have a mobile phone or tablet from my work that is in working order [ ] → Q 12 then 14
    I have a mobile phone/tablet from my work but it is not currently working [ ] → skip to Q 13
    I don't currently have a mobile phone/tablet from my work but I have in the past [ ] → skip to Q 13
    I have never had a mobile phone/tablet provided by my work [ ] → skip to Q 14

12. *If you currently have a functioning mobile phone/device provided by your work, ANSWER ALL THESE QUESTIONS:*
    (a) How many mobile phones provided by your work are you currently using? [___]
    (b) How many tablets provided by your work are you currently using? [___]
    (c) In which year did you receive your [first] mobile device from your workplace? [20___]
    (d) Is any of these devices provided by your work **internet-enabled** *(can use WhatsApp, Facebook, etc.)*? [Y/N]

13. *If you do not currently have a working mobile device provided by your work, but have had in the past, ANSWER ALL THESE QUESTIONS:*
    (a) In which year did you get your [first] mobile device from your work? [20___]
    (b) What happened to this mobile device(s)? *[Tick all that apply]*

        Got damaged [ ]
        Got lost [ ]
        Got stolen [ ]
        Given away [ ]
        Other [ ] → specify ____

    (c) For how long have you been without a mobile device from workplace)? [____] wks / mths / yrs

14. Do you currently own a **personal mobile phone/device** (not provided by your work)? Is it in working order? Have you ever owned one? *[Tick one]*

    I currently have a personal mobile phone/device in working order [ ] → Q 15 then 17
    I have a mobile phone/device but it is not currently working [ ] → skip to Q 16
    I do not currently have a personal mobile phone/device but have in the past [ ] → skip to Q 16
    I have never owned a personal mobile phone/device [ ] → skip to Q 17

15. *If you currently have a personal mobile phone/device in working order, ANSWER ALL THESE QUESTIONS:*
    (a) How many personal mobile phones do you currently own? [___]
    (b) How many personal tablets do you currently own? [___]
    (c) Is any of your personal phones/devices internet-enabled *(can use WhatsApp, Facebook, etc)*? [Y/N]
    (d) Do you currently use your personal phone/device for any aspect of your work as a HEW? [Y/N]
    [Prompt: any aspect includes contacting patients, clients, volunteers, colleagues, etc.]

16. *If you no longer have a personal mobile phone/device in working order but have done in the past, ANSWER ALL THESE QUESTIONS*:

(a) What happened to your phone(s)/device(s)? *[Tick all that apply]*

Got broken [ ]
Got lost [ ]
Got stolen [ ]
Given away [ ]
Other [ ] → specify ____

(b) For how long have you been without a mobile phone/device? [_____] weeks / months / years

(c) Have you ever used a personal phone/device in your work as an HEW? [Y/N]

17. Have you ever received any of the following equipment by your work to facilitate your use of a mobile phone or tablet? [*Tick all that apply and indicate which ones are still working.*]

(a) Solar charger [Y/N] → still working? [Yes/No/NA]
(b) Power bank [Y/N] → still working? [Yes/No/NA]
(c) Other equipment [Y/N] → specify _____________________ → still working? [Yes/No/NA]

Major filter questions: probe carefully to ensure they are answered correctly

18. **CHECK**: Does the respondent currently use a mobile phone or device of any kind in their work as a HEW? [*Prompt to include contacting patients, colleagues, etc., using WhatsApp groups, etc.*]

Yes [ ] → Sections C, D and F (omit Section E)
No [ ] → Q 19

19. **CHECK:** Have the respondent ever used a mobile phone or device of any kind in their work as a HEW? [*Prompt as above and tick one*]

Yes – within the last 4 weeks [ ] → Sections C, D and F (omit Section E)
Yes – within the last 12 months [ ] → Sections D, E and F (omit section C)
Yes – but longer than 12 months ago [ ] → Sections E and F (omit Sections C & D)
No – never [ ] → Sections E and F (omit Sections C & D)

Section C: Use of mobile devices (phone/tablet) in community healthcare

This section is only for respondents who are currently using any mobile device for work-related purposes, or who have done so in the last 4 weeks.

20. **CHECK:** How many mobile devices is the respondent currently using for work-related purposes? [____]

21. **CHECK:** Which is the main mobile device used for work-related purposes? *[Tick one]*
    - Mobile phone provided by work [ ]
    - Tablet provided by work [ ]
    - Your own, personal mobile phone [ ]
    - Your own, personal tablet [ ]
    - Another device (colleague's, friend's…) [ ]

*****From this point, if the respondent does not have/use a tablet, for the rest of the questionnaire refer to 'mobile phone' instead of 'mobile device'.***

22. **CHECK:** Is the phone/device used for work internet-enabled? [Y/N]
    [*If the respondent is not sure, ask to see the phone or ask if it uses data bundles or can do WhatsApp, Facebook, etc.*]

23. **ASK: How often do you currently use a mobile phone/device in your work as an HEW? [*tick one*]**
    - Every day / most days [ ]
    - At least once a week [ ]
    - At least once a month [ ]
    - Rarely or never [ ]

Section C(i): 'Official' Apps and Functions

24. Has your work ever provided dedicated Apps/functions/tools for you use as a HEW? If so, are you still using any of them?
    - Yes, and still using some/all of them [ ] → Q 25
    - Yes, but not currently using any of them [ ] → skip to Q 33
    - No – I have never had these [ ] → skip to Q 33

25. Which of these Apps/functions/tools provided by work are you currently using?
    [*Tick each one that has been used within the last 4 weeks*]
    - Tools for managing/reporting child health & vaccinations [ ]
    - Tools for managing/reporting maternal and new-born health [ ]
    - Tools for managing/reporting Family Planning [ ]
    - Tools for managing/reporting infectious disease outbreaks [ ]
    - Tools for managing medicine supply [ ]
    - Tools for collecting other data [ ]
    - Tools for sending / uploading reports [ ]
    - Toll-free calls/messages (e.g. to call centre) [ ]
    - Any other tools/apps/functions → specify ________________ [ ]

26. Do any of these Apps/tools require <u>data</u> on your mobile phone/device to work? [*Tick one*]

Yes [ ] → proceed to Q 27

No [ ] → skip to Q 29

27. Does your work cover the cost of data to use these? [*Tick one*]

My workplace covers <u>all</u> the data costs of using these functions [ ] → skip to Q 29

My workplace provides a <u>data allowance</u> – I have to pay if I exceed this [ ] → proceed to Q 28

<u>I have to pay</u> for all the data required [ ] → skip to Q 29

28. What data allowance do you get per month? [_____] MB

→ (a) Do you usually exceed this amount? [Y/N]

29. Do any of these Apps/tools that you use require <u>balance</u> (airtime) on your phone/device? [*Tick one*]

Yes [ ] → proceed to Q 30

No [ ] → skip to Q 32

30. Does your work cover the cost of airtime (balance) to use these? [*Tick one*]

My workplace covers <u>all</u> the airtime costs of using these functions [ ] → Q 32

My workplace provides an <u>airtime allowance</u> – I have to pay if I exceed this [ ] → Q 31

<u>I have to pay</u> for all the airtime required [ ] → Q 32

31. What airtime (balance) allowance do you get per month? [_____] Birr

→ (a) Do you usually exceed this amount? [Y/N]

32. *<u>Only for respondents using a mobile phone/device provided by their work (otherwise tick N/A):</u>*

Is your phone/device restricted for these functions only, or it be used for other purposes? [*Tick one*]

Restricted for these specific functions only [ ]

Available for other work-related purposes *but not* personal use [ ]

Available for other work-related purposes *and* personal use [ ]

N/A – Not using a device provided by work [ ]

Section C(ii): "Unofficial" phone use for health extension work

33. Which of the following functions do you use on your phone/device for work related purposes? [*Tick all that you have used within the last 4 weeks for work-related purposes. Do not include any Apps/functions provided by your work*]

a) Voice calls [making or receiving] [ ]
b) SMS (to communicate with patients, fellow HEWs, HDAs, supervisor, etc.) [ ]
c) SMS to send reports, data or information [ ]
d) WhatsApp, Viva, Imo (or similar) to send reports, data or information [ ]
e) WhatsApp, Viva, Imo (or similar) to contact someone (e.g. patient, colleague) [ ]
f) Participating in a WhatsApp (or Imo/Viva) Group (e.g. of fellow HEWs) [ ]
g) Establishing a WhatsApp (or Imo/Viva) Group (e.g. of fellow HEWs) [ ]
h) Facebook or other networking site to seek information [ ]
i) Google (or other internet search) to get information [ ]
j) Downloaded health-related Apps (e.g. from Google Playstore) [ ]
k) Notepad (or similar) for making notes [ ]
l) Camera/video: Taking pictures or film of activities, events, etc. [ ]
m) Camera: Taking shots of reports / paperwork [ ]
n) Camera: Taking pictures of patient symptoms (to seek advice) [ ]
o) Voice recording (e.g. for recording meetings or any other purpose) [ ]
p) Calculator: for collecting data or making reports [ ]
q) Calculator: calculating medicine dosages [ ]
r) Torch to work in the night [ ]
s) Torch for patient examination [ ]
t) Clock or stopwatch/timer (e.g. for taking pulse or breathing rate) [ ]
u) Mobile money or banking (e.g. to collect/send payments or allowances) [ ]
v) Other → specify ____ [ ]

Section C(iii): Mobile phone/device communication in community health work

For the questions in this section, include any form of phone-based communication: calls, SMS, Whatsapp, etc.

34. Do you communicate with patients/clients using a mobile phone/device? [*Tick one*]
[Include any communication including calls, SMS, Whatsapp, etc.]

Yes [ ] → proceed with Q 35
No [ ] → skip to Q 41

35. How often on average do you contact patients/clients using your mobile phone/device? [*Tick one box.*]

Every day / most days [ ]
At least once a week [ ]
At least once a month [ ]
Rarely or never [ ]

36. For what <u>purposes</u> do <u>you contact patients/clients</u> by mobile phone/device? *[List and tick all that apply within the last 4 weeks]*

To arrange visits / appointments [ ]
To check up on them (e.g. medication adherence) [ ]
To report test results (e.g. malaria, TB, HIV, etc.) [ ]
To respond to a call-me-back or missed call [ ]
Social call / greetings [ ]
Other reasons → specify ___________________________ [ ]
None [ ]

37. How often on average do <u>patients/clients contact you</u> on your mobile phone/device? [*Tick one*]

Every day / most days [ ]
At least once a week [ ]
At least once a month [ ]
Rarely or never [ ]

38. For what <u>purposes</u> do <u>patients/clients contact you</u> on your mobile phone/device? *[List and tick all that apply within the last 4 weeks]*

To request or arrange visits / appointments [ ]
To ask for advice [ ]
To request help/transport in an emergency (including childbirth) [ ]
Because they have run out of medication [ ]
Social call / greetings [ ]
Other reasons → specify ___________________________________ [ ]
None [ ]

39. Approximately how many <u>clients'/patients' contacts</u> do you have on your mobile phone/device? [____]

40. Approximately how many <u>clients/patients do you think have your personal contact</u>? [____]

41. <u>Do you communicate with HDAs or other community leaders/volunteers</u> using a mobile phone/device? [*Tick one*]

Yes [ ] → proceed with Q 42
No [ ] → skip to Q 48

42. How often on average do <u>you contact HDAs, etc.</u> using your mobile phone/device? [*Tick one*]

Every day / most days [ ]
At least once a week [ ]
At least once a month [ ]
Rarely or never [ ]

43. For what <u>purposes</u> do <u>you contact HDAs, etc.</u> by mobile phone/device? *[List and tick all that apply within the last 4 weeks]*

Arranging / cancelling / confirming visits, meetings or other activities	[ ]
Following up on specific patients or clients	[ ]
Responding to a call-me-back or missed call from a volunteer	[ ]
Organising an emergency response	[ ]
Social call / greetings	[ ]
Other reasons → specify ____	[ ]
None	[ ]

44. How often on average do <u>HDAs, etc. contact you</u> on your mobile phone/device? [*Tick one*]

Every day / most days	[ ]
At least once a week	[ ]
At least once a month	[ ]
Rarely or never	[ ]

45. For what <u>purposes</u> do <u>HDAs, etc.</u> contact you on your mobile phone/device? *[List and tick all that apply within the last 4 weeks]*

To arrange / cancel / confirm visits, meetings or other activities	[ ]
To give feedback / update on a patient/client	[ ]
To give feedback on an activity or event	[ ]
To request advice	[ ]
For help/transport in an emergency (including childbirth)	[ ]
Social call / greetings	[ ]
Other reasons → specify ____	[ ]
None	[ ]

46. Approximately how many HDA/volunteers' contacts do you have on your mobile phone/device? [___]

47. Approximately how many HDA/volunteers do you think have your personal contact? [___]

48. Do you communicate with your <u>supervisor/boss</u> using a mobile phone/device? [*Tick one*]

Yes	[ ]	→ proceed with Q 49
No	[ ]	→ skip to Q 53

49. How often on average do you contact your supervisor/boss using a mobile phone/device? [*Tick one*]

Every day / most days	[ ]
At least once a week	[ ]
At least once a month	[ ]
Rarely or never	[ ]

50. For what purposes do you contact your supervisor(s)/boss by mobile phone/device? *[List and tick all that apply within the last 4 weeks]*

- To ask for advice or assistance [ ]
- To get help or transport in an emergency [ ]
- To manage supplies of medicines/equipment [ ]
- To send reports / data [ ]
- To send information on specific patients/clients [ ]
- To arrange meetings [ ]
- To report your absence from work [ ]
- To respond to a call-me-back or missed call [ ]
- Social call / greetings [ ]
- Other → specify__________________________ [ ]
- None [ ]

51. How often on average does your supervisor(s)/boss contact you on your mobile phone/device? [*Tick one*]

- Every day / most days [ ]
- At least once a week [ ]
- At least once a month [ ]
- Rarely or never [ ]

52. For what purposes does your supervisor(s)/boss contact you on your mobile phone/device? *[List and tick all that apply within the last 4 weeks]*

- To give instructions / orders [ ]
- To request reports or data [ ]
- To request feedback on a patient/client [ ]
- To request other information [ ]
- To give you information / feedback [ ]
- To arrange meetings [ ]
- For supervision [ ]
- To give feedback on your performance [ ]
- Social call / greetings [ ]
- Other → specify____________________ [ ]
- None [ ]

53. Do you communicate with your fellow HEWs (or other health-workers) using a mobile phone/device?
[*Include peers, junior colleagues and senior colleagues, but not your supervisor/ boss. Tick one.]*

- Yes [ ] → proceed with Q 54
- No [ ] → skip to Q 57

54. How often on average do **you** contact your fellow HEWs/health-workers using a mobile phone/device? [*Tick one*]

- Every day / most days [ ]
- At least once a week [ ]
- At least once a month [ ]
- Rarely or never [ ]

55. How often on average do **your fellow HEWs**/health-workers contact you on your mobile phone/device? [*Tick one*]

Every day / most days [ ]
At least once a week [ ]
At least once a month [ ]
Rarely or never [ ]

56. For what purposes are you in contact your fellow HEWs/health-workers by mobile phone/device? *[Includes contacts in both directions. List and tick all that apply within the last 4 weeks]*

To request/provide advice or assistance [ ]
To request/provide help in an emergency [ ]
To manage supplies of medicines/equipment [ ]
To arrange meetings [ ]
To request/provide information on meetings, training, etc. [ ]
To request/give feedback or information on particular patients/clients [ ]
For supervision [ ]
To respond to a call-me-back or missed call [ ]
Social call / greetings [ ]
Other → specify____ [ ]
None [ ]

57. Have you ever used your phone/device to call emergency transport (e.g. ambulance, taxi, bajaj, etc.) for a health emergency (including childbirth) If so, when? *[Tick one]*

Yes – within the last four weeks [ ]
Yes, but longer than 4 weeks ago [ ]
No, never [ ]

58. How often on average do you **receive** 'call-me-backs' or missed calls in your HEW capacity? [*Tick one*]

Every day / most days [ ] → proceed to Q 59
At least once a week [ ] → proceed to Q 59
At least once a month [ ] → proceed to Q 59
Rarely or never [ ] → Skip to Q 62

59. Who sends 'call-me-backs' (or missed calls) in your HEW capacity? [*Tick all that apply*]

Patients / clients [ ]
HDA/volunteers [ ]
Your supervisor/boss [ ]
Other HEWs/health-workers [ ]

60. Of these, who sends 'call-me-backs' or missed calls you **most often**? [*Tick one*]

Patients / clients [ ]
HDA/volunteers [ ]
Your supervisor/boss [ ]
Other HEWs/health-workers [ ]

61. How often are you able to respond quickly to these call-me-backs or missed calls? [*Tick one*]

Always or almost always [ ]
Most of the time, if I have balance [ ]
Sometimes, if I have balance [ ]
Rarely or never [ ]

62. Do you ever receive work-related calls (from patients, HDAs, colleagues, etc.) outside normal working hours? If so, how often? *[Tick one]*

Every day / most days [ ] → proceed to Q 63
At least once a week [ ] → proceed to Q 63
At least once a month [ ] → proceed to Q 63
Rarely or never [ ] → Skip to Q 65

63. Do you ever receive work-related calls in the night? If so, how often? *[Tick one]*

Every day / most days [ ] → proceed to Q 64
At least once a week [ ] → proceed to Q 64
At least once a month [ ] → proceed to Q 64
Rarely or never [ ] → Skip to Q 65

64. Who contacts you outside of working hours or in the night (for work purposes)? [*Tick all that apply*]

Patients / clients [ ]
HDA/volunteers [ ]
Your supervisor/boss [ ]
Other HEWs/health-workers [ ]

65. Who is most likely to call you outside of working hours or in the night? *[Tick one]*

Patients / clients [ ]
HDA/volunteers [ ]
Your supervisor/boss [ ]
Other HEWs/health-workers [ ]

Section C(iv): Time, costs, data, privacy

66. On an **average working day**, can you estimate how much **time** you spend on a mobile phone/device for work-related purposes? ____ h ____ m

67. In an **average week**, can you estimate how much money you spend on airtime (balance) for work-related purposes? _________ Birr

68. In an average week, can you estimate how much money you spend on data (bundles) for work-related purposes? _________ Birr

69. Do you receive any compensation / allowance to cover these costs (airtime and data bundles)? [Y/N]

→ If yes:

(a) How much airtime (balance) did you receive for the last month? _______ Birr
(b) How much data did you receive for the last month? ________ MB

70. On average, do you spend more money on work-related phone use or personal phone use? [*Tick one*]

- I spend more money for work use than personal use [ ]
- I spend about the same amount for work use and personal use [ ]
- I spend more money for personal use than work use [ ]

71. Does anyone else sometimes use your phones/devices (the ones you use for work)? [*Tick all that apply*]

- Yes – fellow health-workers [ ]
- Yes – friends, family, etc. [ ]
- No [ ]

72. Do you currently have any confidential patient data or images stored on your phone(s)/device(s)? *[Tick all that apply]*

- Yes – I have images of patients [e.g. symptoms] [ ] → continue to Q 73
- Yes – I have other patient data [ ] → continue to Q 73
- No – no patient images/data currently on phone[ ] → skip to Q 74

73. If you currently have confidential patient data/images on your mobile phone/device, are these stored securely (password, log-in, etc.)? *[Tick one only]*

- Yes – all of them are stored securely [ ]
- No – some or all are not all stored securely [ ]

74. Have you ever received any training on the use of mobile phones/devices in your work? If so, what training? *[Tick all that apply]*

- Yes – training on using a phone/device provided by workplace [ ]
- Yes – training on using specific apps/tools [ ]
- Yes – training on using a personal mobile phone/device for work purposes [ ]
- Yes – any other training on mobile phone/device use [ ]
- No – no such training received [ ]

75. Does your workplace have any policies, agreements or codes of practice regarding mobile phone/device use? If so, which ones? *[Tick all that apply]*

- Policies restricting use of phones/devices during work-time [ ]
- Policies about use of personal phones/devices for work-related purposes [ ]
- Policies about storing patient data securely on a phone/device [ ]
- Policies regarding reimbursement for work-related phone/device use [ ]
- Agreements on appropriate use of work-provided mobile phone/device [ ]
- Agreements regarding loss, theft or damage to work-provided phone/device [ ]
- Other → specify ____ [ ]
- No – no policies, agreements or codes of practice [ ]
- Don't know [ ]

Section D: Evaluations of the experience of using mobile phones/devices for **work-related purposes**

Section only for those who have used a mobile device for work-related purposes within the last 12 months

76. Please indicate which of the following benefits you have experienced personally using a mobile phone/device for work-related purposes, and how important these have been.

Possible benefit	*Tick one box per question*		
	**Not identified**	**Small benefit**	**Major benefit**
a. Reduction of workload			
b. Making work more efficient			
c. Completing work faster			
d. Less need to travel			
e. Better communication with patients/clients			
f. Better communication with HDAs / other volunteers			
g. Better communication with fellow health-workers or supervisor			
h. Making work more enjoyable			
i. Making work less stressful			
j. Easier to follow up on patients			
k. Reduces risk of patients defaulting on medication (e.g. HIV, TB)			
l. Improves patient health outcomes			
m. Improves patient confidentiality			
n. Improves medicine supply			
o. Timely submission of data / reports			
p. Easier to arrange meetings / other work logistics			
q. Other → specify:			

77. Has using a mobile phone/device ever saved a patient's life? [Y/N/don't know]

78. Please indicate which of the following difficulties/challenges **you have experienced personally** when using a personal mobile phone/device for work-related purposes, and how serious they are.

Possible difficulty/challenge	*Tick one box per question*		
	**Not identified or N/A**	**Minor challenge**	**Major challenge**
a. Problems with phone network			
b. Problems getting internet			
c. Cost of airtime/balance			
d. Cost of data (bundles)			
e. Battery charge problems			
f. Damage to phone/device			
g. Theft or loss of phone/device			
h. Others (patients/clients/HDA) **not** having phones			
i. Phone disturbing regular work			
j. Too many calls out of work time			
k. Too many calls in the night			
l. Unwanted/irrelevant calls			
m. Negative impacts on marital or social relationships			
n. Risk to patient privacy/confidentiality			
o. Increased workload			
p. Makes work more stressful			
q. Other → specify			

79. Have mobile phones/devices increased or decreased face-to-face meetings with patients/clients? [*Tick one only*]

Increased face-to-face meetings with patients/clients [ ]
Decreased face-to-face meetings with patients/clients [ ]
No change [ ]

80. Have mobile phones/devices increased or decreased face-to-face meetings with your fellow health-workers and supervisors/boss? [*Tick one only*]

Increased face-to-face meetings with HEWs, boss, etc. [ ]
Decreased face-to-face meetings with HEWs, boss, etc. [ ]
No change [ ]

81. Have you ever been **unable** to use your mobile phone/device for work when you wanted or needed to? [Y/N] → If no, skip to Q 82.

→ If yes *ASK ALL THESE QUESTIONS*:

(a) For what reasons has this happened? [*Tick all that apply within the last twelve months*]

Battery out of charge [ ]
No phone signal/network [ ]
No balance (airtime) [ ]
Phone not working properly [ ]
Other: specify _______________ [ ]

(b) For how long were you unable to use a phone/device for your work? [*Tick all that apply*]

Short-term (a few hours or the occasional day) [ ]
Medium-term (several days at a time) [ ]
Longer-term (several weeks or months) [ ]

(c) Has this had any significant consequences on your work? If so, what? [*Tick all that apply*]

No significant consequences [ ]
Negative impact on patient health [ ]
Wasted time (e.g. travelling unnecessarily) [ ]
Missed meeting or training opportunity [ ]
Disappointed clients/patients/HDAs by missing an appointment/meeting [ ]
Disappointed fellow-health workers or supervisor by missing a meeting [ ]
Other → specify ____ [ ]

(d) To your knowledge, has your inability to use a mobile phone/device when needed ever resulted directly in a patient's death? [*Tick one*]

Yes [ ]
No [ ]
Don't know [ ]

82. In the last four weeks, have you had to make any sacrifices (financial or other) in order to use a mobile phone/device for work-related purposes? [Y/N]

→ If yes, what? *[tick all that apply]*

Food or other essentials	[ ]
Non-essentials	[ ]
Savings	[ ]
Other ____________________	[ ]

83. How easy/difficult would it be for someone to do your job without using a mobile phone/device? *[Tick one]*

Easy	[ ]
Quite difficult	[ ]
Very difficult	[ ]
Impossible	[ ]

84. *For those using internet:* How easy/difficult would it be for someone to do your job without the internet on your phone/device? *[tick one]*

Easy	[ ]
Quite difficult	[ ]
Very difficult	[ ]
Impossible	[ ]
N/A (don't use internet)	[ ]

85. *For those without (or not using) internet:* Does not having/using the internet make any aspects of your work more difficult?

Yes	[ ]
No	[ ]
N/A (internet user)	[ ]

(a) → If yes, which aspects are more difficult? *[Tick all that apply]*

Keeping in touch with patients/clients or following up on them	[ ]
Keeping in touch with fellow health-workers and/or supervisor	[ ]
Responding in emergencies	[ ]
Being aware of current disease outbreaks /other important information	[ ]
Arranging meetings	[ ]
Being aware of meetings and training opportunities	[ ]
Collecting data	[ ]
Compiling and/or sending reports	[ ]
Other: specify → ____	[ ]

Section E: Non-use of a mobile phone for work.

This section is only for those who do not use mobile phone/device for work (and who have not done so within the last four weeks).

86. Check: Have you ever used a mobile phone/device in your work as a HEW?

    Yes [ ] → proceed to Q 87

    No [ ] → skip to Q 89

87. How long ago did you stop using a mobile phone/device in your work? _______ months / years

88. Why did you stop using a mobile phone/device for your work? *[Tick all that apply.]*

    Phone got damaged [ ]

    Phone got lost [ ]

    Phone got stolen [ ]

    Too expensive [ ]

    Network problems [ ]

    Other reason [ ] → please state ___________________________________

89. Does not using a mobile phone/device make any aspects of your work more difficult?

    Yes [ ] →

    No [ ]

    (a) → If yes, which aspects are more difficult? *[Tick all that apply]*

    Keeping in touch with patients/clients and following up on them [ ]

    Keeping in touch with fellow health-workers and/or supervisor [ ]

    Responding in emergencies [ ]

    Being aware of current disease outbreaks /other important information [ ]

    Arranging meetings [ ]

    Being aware of meetings and training opportunities [ ]

    Collecting data [ ]

    Compiling and/or sending reports [ ]

    Other: specify → ____ [ ]

90. To your knowledge, has not having a phone ever led to a patient's health deteriorating? [*Tick one*]

    Yes [ ]

    No [ ]

    Don't know [ ]

91. To your knowledge, has not having a phone ever led to a patient's death? [*Tick one*]

    Yes [ ]

    No [ ]

    Don't know [ ]

92. Are there any *advantages* to not using a mobile phone for your work? *[Tick all that apply]*

Yes – patients or HDAs are not disturbing me with their calls/requests [ ]
Yes – supervisor/boss or fellow HEWs are not disturbing me with their calls/requests [ ]
Yes – I don't have to use my money on airtime / data for work [ ]
Yes – I don't have to worry about charging, network, etc. [ ]
Yes – other: → specify ____ [ ]
No – no advantages [ ]

93. Have you come under any pressure to get/use a mobile phone for your work? [Y/N]

(a) → If yes, from whom? *[Tick all that apply]*

From patients/clients/HDAs [ ]
From fellow health-workers [ ]
From manager/supervisor/boss [ ]
Other: specify _______________ [ ]

Section F: Ways forward

This section is for EVERYONE.

94. Which of the following initiatives would help you to use a mobile phone (or other device) more effectively in your work as a HEW? Add your own if you have other ideas

    Make it clear that we are not in a position to provide any of these

**Initiative**	*Tick if this would be beneficial to you*
a. Provide a mobile phone for work use	
b. Upgrade my basic phone to an internet-enabled or smart phone	
c. Provide dedicated SIM card for work use	
d. Provide free calls and SMS	
e. Provide a monthly airtime allowance	
f. Provide a monthly data bundle phone allowance	
g. Provide a good-quality solar phone charger	
h. Provide a good-quality power bank	
i. Provide training on using phones for work	
j. Provide a dedicated helpline for advice / emergency assistance	
k. Other → specify	
l. Other → specify	
m. Other → specify	

95. **Which <u>three</u> of these initiatives would be the <u>most beneficial</u>?** In order of importance:
    [*State the letter in each case. If fewer than three are indicated, leave blanks*]

    (a) Most important: [ ] *[Indicate letter]*
    (b) Second most important: [ ] *[Indicate letter]*
    (c) Third most important: [ ] *[Indicate letter]*

END OF QUESTIONNAIRE.

Thank the respondent and ask whether they have any further questions or comments.

IMAGINE (Informal Mhealth in Africa: Grassroots Initiatives & Networks)

FOCUS GROUP GUIDE FOR COMMUNITY HEALTH WORKERS

Date: DD / MM / YY   Facilitator's name ______________________

Time started____________   Note-taker's name _____________________

Time finished__________   Settlement identifier ___________________

Country: [Ghana / Ethiopia / Malawi]   Health centre catchment area ____________

Audio file number & device [where consent is given for recording] __________

1) Introductions [see separate documents]

- Explain the purpose of the research and this FDG.
- Check informed consent and explain importance of confidentiality.
- Ask each participant to introduce themselves and complete table below
- Request permission to audio record the FGD.

Participant	Job/role (e.g. HSA)	Gender	Age	Educational / training qualification	Length of service as CHW	Mobile phone ownership
1						
2						
3						
4						
5						
6						
7						

2) Explore the work being done by community health workers:

- What are your main tasks and responsibilities?
- What training and support have you received / do you receive?
- What challenges do you experience in your work?
- What factors facilitate your work?

3) Work phones and 'formal mhealth'

- Does anyone have access to official work-based phones (mobiles, landlines, 2-way radios)?
- Has anyone been involved in any official mhealth initiatives?
- Details and experiences of either/both?

4) Personal mobile phones and CHW work: what, how, why?

- Find out who has a mobile phone and who has internet-enabled phones.
- Do you use your personal mobile phones for work?
    - How often do you use them?
    - How / for what purposes? [Prompts: contacting colleagues (when & why?), contacting clients/patients (when & why?), reporting, weekly, monthly, and

quarterly performances, getting feedbacks from immediate boss, supervisors….., Managing logistics, getting information, getting help in emergencies, etc.
- *Encourage participants to share their personal experiences (good and bad) and stories without compromising client/patient confidentiality. How frequent?*

5) Phonelessness
- Do any participants *not* have [or not have access to] a personal mobile phone?
- Are you aware of any CHWs who do not have personal mobile phones?
- Why do some CHWs not have personal mobile phones? Cost? Interest?
    - What are their experiences? What (if any) challenges have they faced?
- To what extent is it *expected* that CHWs will have and use personal phones in their work?
- Are there any opportunities not available to CHWs without personal mobile phones?
- Do CHWs without phones come under pressure to get one? [From whom, why, etc.?]

6) Evaluations of experiences
- What have been the benefits/advantages of using personal mobile phones in your work?
    - Prompts: keeping in touch better with clients, patients, colleagues, supervisors, etc.; able to manage work logistics better; use in emergencies, etc.
- What (if any) have been the difficulties of using personal mobile phones in your work
    - Prompts: air time, phone charge, phone signal, phone repair, network coverage, etc.
- What (if any) have been the disadvantages of using personal mobile phones in your work?
    - Prompts: cost, increased workload, decreased face-to-face contact with clients, etc.
- How do you weigh up and evaluate these benefits, drawbacks and challenges?

7) Ways forward
- Have there been any initiatives to address the difficulties/challenges identified above?
    - Either using phones or not having access to phones, etc.
- What are the most important steps that could be taken to address any problems identified?
    - By government, by managers/bosses, by CHWs, by clients/patients?
    - How should these be prioritised?

8) Conclusion
- Ask whether participants have anything else they would like to contribute.
- Thank participants and remind them of responsibilities vis-à-vis confidentiality.

Notes and reflections: *How did the FGD go? Interruptions? Participants engaged? Any problems? Any areas they were especially keen to or unwilling to talk about?*

IMAGINE (Informal Mhealth in Africa: Grassroots Initiatives & Networks)

FOCUS GROUP GUIDE FOR CLIENTS

Date: DD / MM / YY — Facilitator's name ______________________

Time started____________ — Note-taker's name _____________________

Time finished__________ — Settlement identifier ___________________

Country: [Ghana / Ethiopia / Malawi] — Health centre catchment area ____________

Audio file number & device [where consent is given for recording] _________

1) Introductions [see separate documents]

- Explain the purpose of the research and this FDG.
- Check informed consent and explain importance of confidentiality.
- Ask each participant to introduce themselves and complete table below
- Request permission to audio record the FGD.

Participant	Gender	Age	Occupation	Mobile phone ownership?
1				
2				
3				
4				
5				
6				
7				

2) Personal mobile phones and healthcare: what, how, why?

- Which participants own and/or have access to a mobile phone? And internet phones?
- Are participants using personal mobiles phones for healthcare-related purposes?
- **How** and **why** are people using mobile phones in in relation to healthcare?
    - Prompts: 'formal' mhealth initiatives, contacting health-workers, reporting performances, receiving feedback, getting information/advice, getting help in emergencies, etc.
- *Encourage participants to share their personal experiences of using mobiles for health care.*

3) Evaluations of experiences

- What have been the benefits/advantages of using personal mobile phones for healthcare?
    - Prompts: able to keep in touch better with heath-workers, able to access information, useful in emergencies, etc.
- What (if any) have been the difficulties of using personal mobile phones for healthcare?
    - Prompts: maintaining air time, phone charge, phone signal, phone repair, network coverage, etc.
- What (if any) have been the disadvantages of using personal mobile phones for healthcare?

    - Prompts: cost, decreased face-to-face contact with health-workers, wrong advice etc.
- Overall, what have been the impacts of using mobile phones on healthcare and wellbeing?
    - Do these vary for different kinds of clients/patients, (e.g. by age, gender, SES, etc.)
- How do you weigh up and evaluate these benefits, drawbacks and challenges?

4) Phonelessness

- Do any participants *not* have [or not have access to] a personal mobile phone?
- Do you know other people in the community without [access to] a mobile phone?
    - What are their experiences?
    - What challenges have they faced in accessing healthcare without a phone?
- To what extent is it *expected* that clients/patients will have and use phones in healthcare?
    - Explain?

5) Ways forward

- Are you aware of any initiatives that have tried to address any of these challenges?
    - Details: which organisation(s)? Where? When? Perceived impact?
- What are the most important steps that could be taken to address any problems identified?
    - By patients/clients?
    - By other (e.g. By government, health-workers, etc.)?
    - How should these be prioritised?

6) Conclusion

- Ask whether participants have anything else they would like to contribute.
- Thank participants and remind them of responsibilities vis-à-vis confidentiality.

Notes and reflections: *How did the FGD go? Interruptions? Participants engaged? Any problems? Any areas they were especially keen to or unwilling to talk about?*
